# Mapping Emergency Medicine Data to the Observational Medical Outcomes Partnership Common Data Model: A Gap Analysis of the American College of Emergency Physicians Clinical Emergency Data Registry

**DOI:** 10.1016/j.acepjo.2024.100016

**Published:** 2025-01-10

**Authors:** Inessa Cohen, Zihan Diao, Pawan Goyal, Aarti Gupta, Kathryn Hawk, Bill Malcom, Caitlin Malicki, Dhruv Sharma, Brian Sweeney, Scott G. Weiner, Arjun Venkatesh, R. Andrew Taylor

**Affiliations:** 1 Department of Emergency Medicine, Yale School of Medicine, New Haven, Connecticut, USA; 2 Section for Biomedical Informatics and Data Science, Yale University School of Medicine, New Haven, Connecticut, USA; 3 Program of Computational Biology and Bioinformatics, Yale University, New Haven, Connecticut, USA; 4 American College of Emergency Physicians, Washington, DC, USA; 5 Department of Emergency Medicine, Brigham and Women’s Hospital, Boston, Massachusetts, USA; 6 Center for Outcomes Research and Evaluation (CORE), Section of Cardiovascular Medicine, Yale School of Medicine, New Haven, Connecticut, USA

**Keywords:** databases, registries, electronic health records, informatics, emergency service, quality of health care, opioid/adverse effects

## Abstract

**Objectives:**

This study aims to conduct a gap analysis to determine the feasibility of mapping electronic health record data from the Clinical Emergency Data Registry (CEDR) to the Observational Medical Outcomes Partnership Common Data Model (OMOP-CDM).

**Methods:**

We employed a structured approach using a custom-built comparison matrix. This matrix facilitated the alignment of CEDR data fields with the corresponding elements in the OMOP-CDM schema. Each field was evaluated for compatibility, with categorization into 3 distinct types: direct matches, fields requiring transformation, and fields with no OMOP-CDM equivalent. The mapping process was informed by consultations with the Observational Health Data Sciences and Informatics community forums and was guided by existing documentation and best practices in data harmonization. We performed descriptive analyses, quantifying the extent of direct matches and identifying the specific transformations needed for each CEDR-CDM field to ensure compliance with the OMOP-CDM model.

**Results:**

Our analysis indicates a high degree of compatibility between CEDR and OMOP, with over 90% (244/269) of CEDR fields being successfully mapped. Specifically, 173 fields had direct matches, whereas 71 required transformations. Challenges identified include addressing fields unique to CEDR with no OMOP-CDM equivalent and managing the transformations required for proper alignment.

**Conclusion:**

The OMOP-CDM presents a promising framework for standardizing emergency medicine data, thereby enhancing future query automation, analytics, and cross-institutional collaboration. Despite the potential challenges in capturing unique CEDR fields and addressing necessary transformations, most emergency department data can be standardized within the OMOP-CDM, fostering broader insights and applications in research and public health.

**The Bottom Line**

This study looked at whether data from the Clinical Emergency Data Registry (CEDR) could be translated into a common format, Observational Medical Outcomes Partnership (OMOP), used by hospitals and research centers. The results show that >90% of CEDR data fields could be directly mapped or adjusted to fit this format. Conversion to OMOP would facilitate interoperable data exchange, the use of automated tools, and enhanced data quality for research and assessment of patient care.

## 1 Introduction

### 1.1 Background

The explosive growth of electronic health care data in medicine offers a unique opportunity at the national level to improve health surveillance, illuminate care disparities, shed light on rare diseases, and aid the development of multifaceted models for various health conditions. Within emergency medicine, the Clinical Emergency Data Registry (CEDR), developed by the American College of Emergency Physicians (ACEP), represents a significant progression in harnessing big data at the national level.^1^ As a collaborative effort involving more than 5,000 emergency departments, CEDR has been instrumental in facilitating data-driven decision making for quality improvement and reimbursement processes. Recognizing the imperative for enhanced data utility, the ACEP Board of Directors, in 2022, authorized CEDR's evolution into the Emergency Medicine Data Institute (EMDI).^2^^,^^3^ This transition is strategic, transforming EMDI into a standardized repository for emergency medicine data, augmenting research capabilities, and enabling a deeper analytical understanding of emergency care.

### 1.2 Importance

The utility of EMDI for research is currently constrained by the lack of a standardized data schema that aligns with existing research practices. Data standardization of CEDR data elements using a common data model (CDM) would not only streamline the data extraction process and automate queries for quality improvement but also allow for better research utility and interoperability. Addressing this gap is critical for enabling the integration of EMDI into the broader ecosystem of existing and emerging research platforms and data analytic tools.

The Observational Medical Outcomes Partnership (OMOP) CDM offers a powerful solution to standardize CEDR data. OMOP is a standardized patient-level database that enables transparent and reproducible observational research in the open science community.^4^ Since its development in 2013, the OMOP-CDM has gained widespread adoption among hospitals and academic medical centers in the United States and worldwide, currently housing data from over 950 million patients across 49 countries.^5^ Commonly used research datasets using OMOP-CDM include the National COVID Cohort Collaborative (N3C),^6^ which integrates COVID-19 data from over 75 institutions, the Medical Information Mart for Intensive Care (MIMIC-IV),^7^^,^^8^ a de-identified intensive care unit database with over 40,000 patients, and the All of Us Research Program,^9^ a precision medicine initiative collecting electronic health record (EHR) and genomic data from one million diverse populations across the U.S. Previous research has also shown success in mapping EHR data to the OMOP-CDM for clinical use, but variation exists by specialty and country.^10^, ^11^, ^12^, ^13^, ^14^, ^15^, ^16^, ^17^, ^18^, ^19^

### 1.3 Goals of This Investigation

Our investigation aims to conduct a comprehensive gap analysis to assess the compatibility of EHR data from CEDR with the OMOP-CDM. The anticipated outcomes of this research are to lay the groundwork for subsequent endeavors to align the EMDI with prevalent data schemas, thereby enhancing interoperability and facilitating a unified platform for emergency medicine research and analytics. This alignment can simplify the integration process, allowing for more efficient participation in CEDR, collaboration across institutions, and enhancing the quality and quantity of research in emergency medicine.

## 2 Methods

### 2.1 Characteristics of the OMOP-CDM Schema

The current version of the OMOP-CDM schema (v 5.4) to date includes 39 tables and approximately 400 fields to capture different aspects of patient-level data.^4^ These data elements are all standardized and curated by the Observational Health Data Sciences and Informatics (OHDSI) community and can be linked to each other using de-identified patient-level and visit-level identifiers. These tables can be classified into 6 high-level categories: standardized clinical data, standardized vocabularies, standardized health systems, standardized health economics, standardized derived elements, and standardized metadata. Most of the OMOP-CDM tables fall into either standardized clinical data or standardized vocabularies. The standardized clinical data includes 17 tables with patient-level characteristics such as demographics, conditions, and measurements. The standardized vocabularies include 10 tables that capture different medical coding system terminologies (LOINC, SNOMED, ICD10) and translate them into concepts and domains. The standardized health system includes 3 tables that contain information about the location of the visit and clinician. The standardized health economics includes 2 tables that capture the costs of any medical event and insurance details. The standardized derived elements contain 5 tables that are specific to the analysis and allow researchers to create a cohort. Finally, the standardized metadata includes 2 tables with metadata information about the transformed data set and the source database.

### 2.2 Characteristics of the CEDR Schema

Of the abstractable fields across EHR and billing feeds, CEDR stores standardized data across 19 tables and 269 fields at the time of the analysis (Supplementary Appendix 1). The structured fields are used for government reporting requirements. Like the OMOP-CDM, the CEDR database is normalized but has information collected at the encounter level. CEDR tables can be classified into 4 categories: billing/payer, encounter, patient, and orders/results. There are 7 tables in the encounter category that contain clinically relevant information at the time of the visit (eg, diagnoses, procedures, etc). The patient category contains 7 tables that capture demographic and historical information (eg, diagnoses, medications, social history, etc). Orders/results contain 2 tables that capture any lab information. Finally, the 2 tables in the billing/payer category are analogous to the standardized health economics table in the OMOP-CDM and capture insurance-related information.

### 2.3 Mapping Procedure

A comparison matrix was created by 2 team members (IC, RAT) in Microsoft Excel to map tables and fields from CEDR to the OMOP-CDM schema (Supplementary Appendix 1). IC holds a Master of Public Health in Epidemiology (MPH) and RAT holds an MD and a Master’s in Health Science (MSHI), both with extensive experience in clinical data mapping and standardization processes. A “field type” column was created to indicate fields that match, fields that require transformation, and fields with no equivalent. The field type was determined by comparing the field descriptions and the type constraints in the OMOP-CDM and referencing documentation for both schemas.

**Figure 1:**
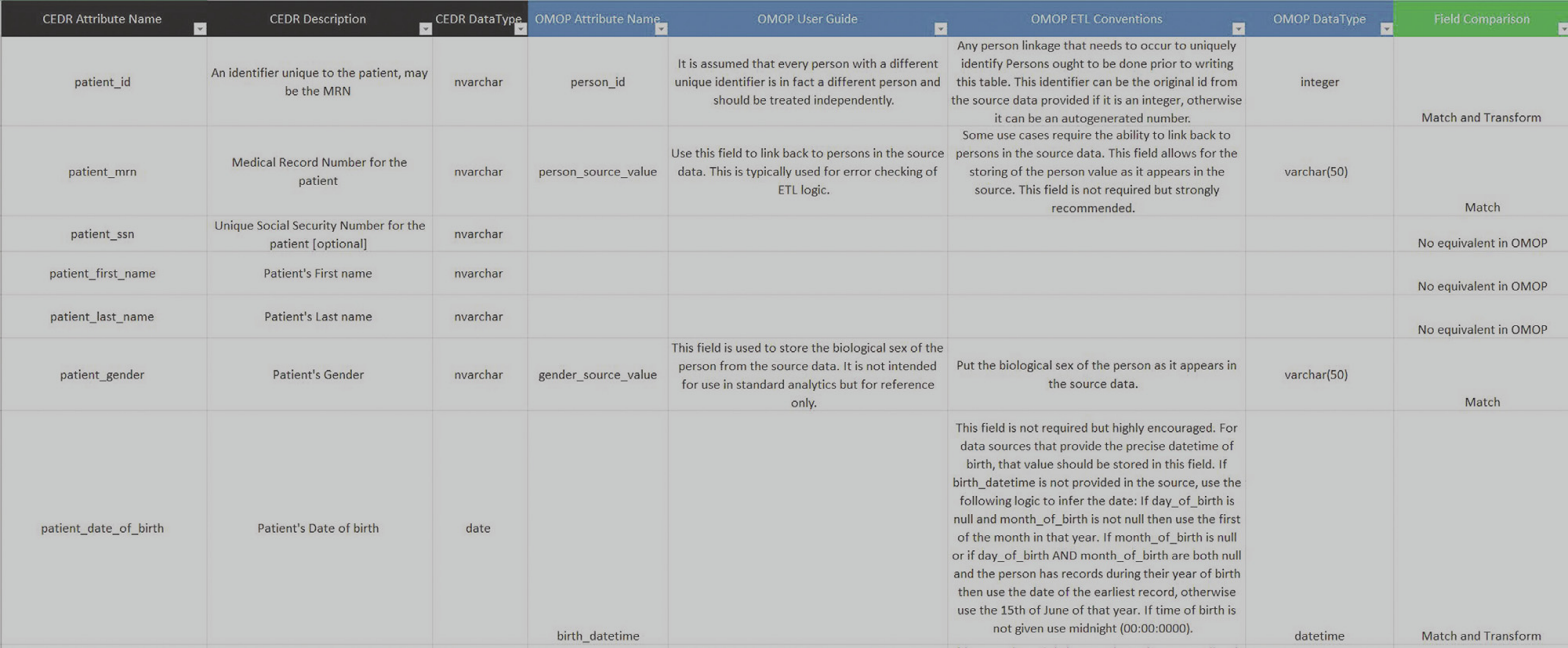
Comparison matrix showing example mapping for the CEDR patient to OMOP Person tables. CEDR, Clinical Emergency Data Registry; OMOP-CDM, Observational Medical Outcomes Partnership Common Data Model.

A match was defined as a variable that had a direct equivalent in the OMOP-CDM based on the field description as well as the data type. For example, “patient_gender” from the CEDR schema in the patient table matched the “gender_source_value” in the OMOP-CDM schema in the Person table (Fig 1).

A transformation was defined as a variable that had a similar field description but may need to be converted into a different data type or would require additional processing (changing format, recoding values to align with OMOP standards, or creating new variables). For example, “patient_date_of_birth” would need to be transformed from a date into a datetime in order to match the “birth_datetime” field in the OMOP-CDM schema (Fig 1).

When values in the CEDR registry did not map to the OMOP-CDM, this was classified as a field that had no equivalent. For example, “patient_ssn,” which stores the social security number of each patient, is unique to CEDR and has no equivalent in the OMOP-CDM (Fig 1).

For fields with multiple potential matches or ambiguity, where a field in the CEDR schema could correspond to multiple OMOP-CDM fields or lacked sufficient description for a direct match, the OHDSI forums^20^ and Athena,^21^ a web-based interface for querying standardized vocabularies in the OMOP-CDM, were referenced to ensure adherence to community conventions. Both team members (IC and RAT) independently reviewed these fields and discussed any ambiguities together. For example, a field with multiple potential matches is “service_location_id” in the CEDR schema, which could correspond to either “location_id” or “care_site_id” in the OMOP-CDM. After review, it was determined that “care_site_id” was the better option. Similarly, an ambiguity arose with diagnosis priority, as there is no field explicitly called “primary diagnosis” or “secondary diagnosis” in the OMOP-CDM. After careful review, it was determined that diagnosis priority could be captured using the “condition_status_concept_id” field with primary diagnosis = “32,902” and secondary diagnosis = “32908.” No formal interrater reliability assessment was conducted, as most fields had direct and clear corresponding columns.

### 2.4 Analysis

**Figure 2:**
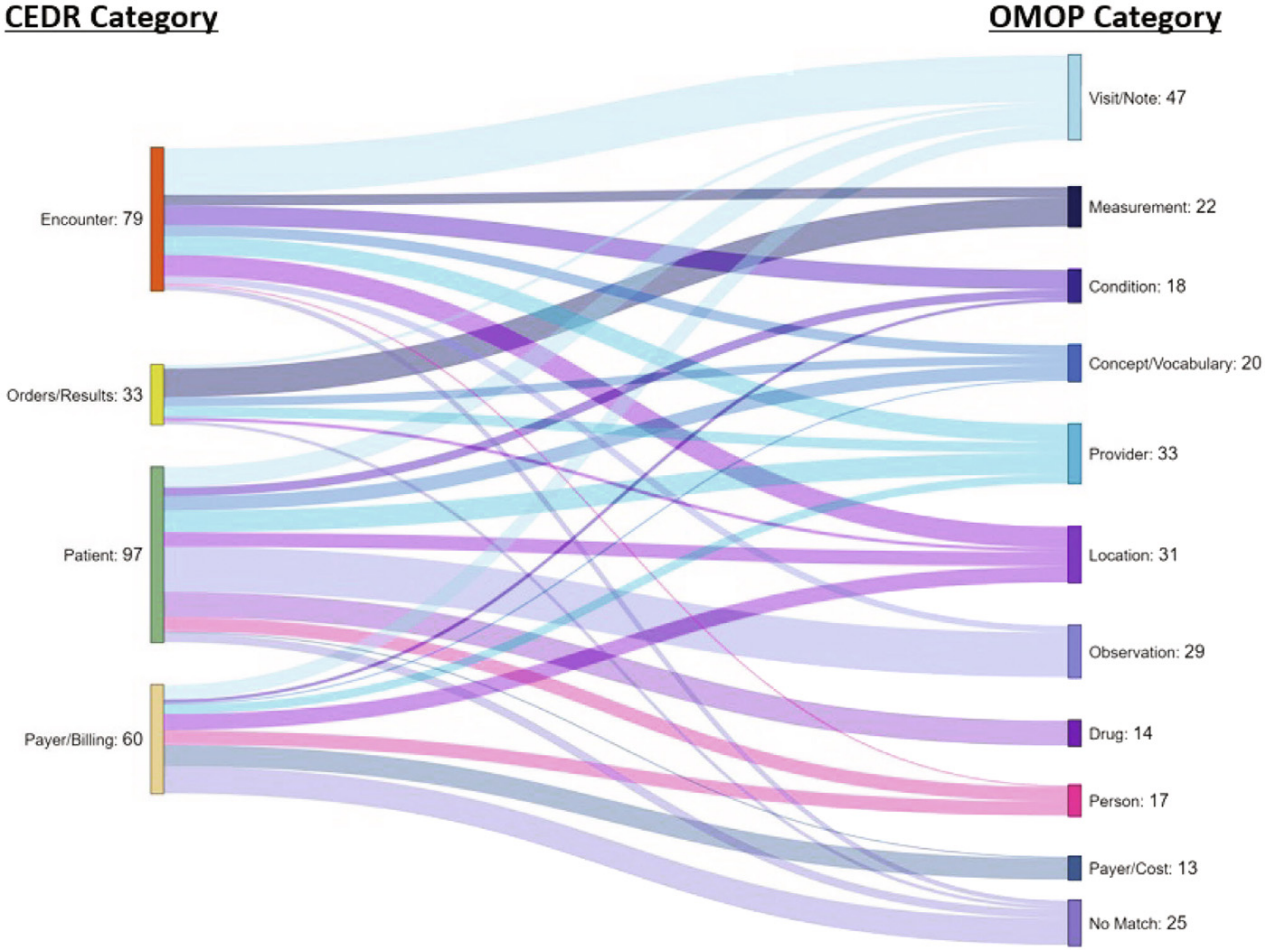
Sankey diagram depicting the proportions of CEDR categories mapped to OMOP-CDM categories. The different colors are used for visual differentiation between mapping paths and do not represent specific categories. CEDR, Clinical Emergency Data Registry; OMOP-CDM, Observational Medical Outcomes Partnership Common Data Model.

We employed standard descriptive statistics to summarize the data. Frequencies and percentages were calculated for categoric variables to provide insights into the distribution of data across different categories. Counts were used to describe the number of occurrences for each category. To visualize the flow and mapping between CEDR and the OMOP-CDM within our data set, we constructed a Sankey diagram (Fig 2).^22^ This diagram effectively illustrates the magnitude of different flows and their relationships, providing a clear visual representation of the distribution and progression of variables. All statistical analyses were conducted using R, and diagrams were created using SankeyMatic (https://sankeymatic.com/).

## 3 Results

A field-by-field analysis comparing the CEDR schema to the OMOP-CDM schema revealed that over 90% (244/269) of the CEDR fields can be mapped successfully to the OMOP-CDM. Among these, 173 were direct matches, and 71 required transformations (Table, Fig 2).

**Table:** Mapping results from CEDR tables to the OMOP-CDM by field type

CEDR table category	Match (N = 173) n (row %)	Match and transform (N = 71) n (row %)	No equivalent (N = 25) n (row %)
Billing/payer			
Billing data (N = 44)	21 (47.7%)	10 (22.7%)	13 (29.5%)
Billing location (N = 6)	3 (50.0%)	3 (50.0%)	0 (0%)
Payer (N = 10)	8 (80.0%)	0 (0%)	2 (20.0%)
Encounter			
Encounter (N = 20)	13 (65.0%)	6 (30.0%)	1 (5.0%)
Encounter diagnosis (N = 16)	10 (62.5%)	5 (31.3%)	1 (6.3%)
Encounter patient complaint (N = 7)	6 (85.7%)	1 (14.3%)	0 (0%)
Encounter procedure (N = 12)	10 (83.3%)	2 (16.7%)	0 (0%)
Location (N = 6)	3 (50.0%)	3 (50.0%)	0 (0%)
Plan of care (N = 9)	9 (100%)	0 (0%)	0 (0%)
Vital signs (N = 9)	9 (100%)	0 (0%)	0 (0%)
Patient			
Allergy (N = 15)	9 (60.0%)	4 (26.7%)	2 (13.3%)
Family history (N = 12)	9 (75.0%)	3 (25.0%)	0 (0%)
Medication (N = 21)	9 (42.9%)	11 (52.4%)	1 (4.8%)
Patient (N = 14)	8 (57.1%)	3 (21.4%)	3 (21.4%)
Patient notes (N = 11)	6 (54.5%)	5 (45.5%)	0 (0%)
Patient problem (N = 10)	6 (60.0%)	4 (40.0%)	0 (0%)
Social history (N = 14)	10 (71.4%)	4 (28.6%)	0 (0%)
Orders/Results			
Orders (N = 14)	11 (78.6%)	2 (14.3%)	1 (7.1%)
Result observation (N = 19)	13 (68.4%)	5 (26.3%)	1 (5.3%)

CEDR, Clinical Emergency Data Registry; OMOP-CDM, Observational Medical Outcomes Partnership Common Data Model.

The CEDR tables that included the highest proportion of direct matches to the OMOP-CDM included fields from the following tables: plan of care (100%, 9/9), vital signs (100%, 9/9), encounter patient complaint (85.7%, 6/7), encounter procedure (83.3%, 10/12), payer (80.0%, 8/10), orders (78.6%, 11/14), and family history (75.0%, 9/12). These fields can be predominantly mapped to the following tables: note, measurement, condition occurrence, procedure occurrence, payer plan period, measurement, and observation, respectively. It is important to note that none of the relationships were one-to-one. Instead, fields from a single CEDR table could map to multiple OMOP-CDM tables depending on the context. For example, although fields from the CEDR Patient CEDR table were predominately mapped to the Person table, they had secondary mappings to other tables (death, care site, payer plan period, and observation).

The CEDR tables with the highest proportion of fields requiring transformations included: medication (52.4%, 11/21), location (50.0%, 3/6), and billing location (50.0%, 3/6). These transformations range in complexity required, such as data type conversions (date to datetime), concatenating fields into one (combining first and last names to create clinician names), and creating new fields using both structured and unstructured data (extracting medication dose and calculating medication strength) (Supplementary Appendix 1).

Finally, 9.5% (25/269) fields in CEDR had no equivalent in the OMOP-CDM. These fields included fields predominately from the patient and payer tables, which included protected health information (name, social security number), canceled laboratories/procedures, billing data details (a condition related to employment, auto accident, or other), or redundant fields containing abbreviations of other fields that were already matched (specialty code and specialty description; specialty code was not mapped, and specialty description retained).

## 4 Limitations

This study is subject to several limitations that should be considered when interpreting its findings. The gap analysis conducted is preliminary and based solely on schema comparisons rather than patient-level data. This approach limits our ability to capture the nuances and complexities that real patient data might present, potentially leading to underestimations or overestimations of the compatibility and necessary transformations between the CEDR and OMOP-CDM schemas. The mapping exercise, although comprehensive, may not have identified all possible matches or required transformations due to the inherent limitations of comparing field descriptions without the context of actual data content. Some fields might be more versatile or have broader applicability in practice than our analysis suggests. Some data transformations required to align CEDR fields with the OMOP-CDM schema may be more complex than anticipated. This complexity can introduce challenges in data integrity, accuracy, and the preservation of clinical meanings. The study does not account for the potential operational and technical difficulties in executing these transformations. The findings from this schema-based analysis may not be generalizable to other emergency medicine databases or other disciplines within health care due to the unique structure and content of the CEDR and OMOP-CDM schemas. The specific challenges and solutions identified may be particular to the context of this investigation. Both the CEDR and OMOP-CDM schemas are subject to updates and revisions. Our analysis is based on the versions available at the time of the study, and future changes in these schemas could affect the relevance and accuracy of our findings. Implementing a standardized data schema across diverse emergency departments that contribute to CEDR involves significant resources, including time, technical expertise, and financial investment. The practicality and feasibility of such implementations were not assessed in this study.

The alignment of CEDR with the OMOP-CDM is a promising advancement for standardizing emergency medicine data, crucial for improving research capabilities, clinical decision making, and public health surveillance. Although challenges remain in accommodating unique data elements and ensuring scalable transformations, the ongoing development of biomedical informatics tools and models in CEDR is essential to fully realize the benefits of a standardized data environment in emergency care.

## 5 Discussion

The rapid expansion of electronic health care data and the development of CEDR have significantly enhanced data-driven decision making in emergency medicine. Transitioning to the EMDI and partitioning a research-ready data set using an OMOP-CDM promises further standardization and interoperability. Our analysis revealed that although a large portion of the CEDR data aligns well with the OMOP-CDM schema, with over 90% of fields either directly matching or amendable via transformation, several challenges in fully integrating these systems remain.

In the broader context of data standardization in health care, our results align with findings from other studies that demonstrate the benefits of adopting the OMOP-CDM for enhanced data interoperability and research flexibility.^10^, ^11^, ^12^, ^13^, ^14^, ^15^, ^16^, ^17^, ^18^, ^19^ For example, previous research within the OHDSI network has successfully utilized the OMOP-CDM to conduct large-scale, multiinstitutional studies, allowing researchers to share code and methodologies seamlessly.^23^ These studies have not only expedited research processes but have also improved data quality and reliability across different health care domains. Comparatively, our study indicates similar potential for emergency medicine, albeit with noted challenges specific to the discipline. The lack of fields for protected health information and the need for custom transformations are significant hurdles that have been observed in other specialties during the initial phases of the OMOP-CDM adoption. However, unlike some disciplines, such as oncology, where high granularity in data are often essential, emergency medicine may find the existing structure of the OMOP-CDM more immediately compatible, contingent on addressing the unique data elements pertinent to emergency care practices.

Looking forward, addressing the identified gaps in CEDR and OMOP-CDM integration will require developing tailored solutions for emergency medicine-specific data fields and transformations. Enhancing the OMOP-CDM schema to accommodate unique CEDR fields, possibly through informatics community-driven extensions, could mitigate some of the integration challenges. Moreover, scalability and the practicality of standardizing diverse data sources across numerous sites call for a phased implementation strategy, potentially incorporating an intermediate data mart to bridge the gap between CEDR and OMOP-CDM schemas. Continuous engagement with the OHDSI community and iterative feedback will be crucial to refine the integration process and maximize the utility of emergency medicine data for research and clinical practice.

The transition of CEDR to the OMOP-CDM has significant practical implications for emergency physicians and future CEDR users. Aligning CEDR with OMOP-CDM facilitates more seamless data integration and sharing across institutions, which can expedite multi-center research efforts, improve data quality, and enhance clinical decision making through standardized data analytics. Emergency physicians can leverage this alignment for automated quality improvement processes, enabling quicker access to actionable insights that can directly improve patient care.

For CEDR users, this transition reduces the burden of data manipulation, allowing quicker participation in research networks using the OMOP-CDM, such as the National COVID Cohort Collaborative (N3C) and other large-scale research consortia. Additionally, it opens new opportunities for collaboration with other institutions already using OMOP-CDM, fostering a broader research ecosystem in emergency medicine. Building on this initial mapping feasibility analysis, future work will focus on piloting the OMOP-CDM schema in emergency departments contributing to CEDR. This includes validating the mapped fields with real-world data to ensure that transformations maintain data integrity and clinical relevance. Further, collaborations with the OHDSI community will be essential for refining the mapping process, addressing specialty-specific data needs, and extending the OMOP-CDM schema to better accommodate emergency medicine’s unique data characteristics.

## Author Contributions

RAT, IC, and AV conceived the study and designed the approach, IC, RAT, and AV drafted the manuscript, and all authors contributed substantially to its revision. RAT and IC take responsibility for the paper as a whole.

## Funding and Support

Supported by the 10.13039/100000026National Institute On Drug Abuse of the 10.13039/100000002National Institutes of Health under HEAL Initiative: HEAL Data2Action – Innovation and Acceleration Projects, Phased Awards. Award 1R61DA059169-01. (Principal investigators: AV, KH, and RAT). The content is solely the responsibility of the authors and does not necessarily represent the official views of the National Institutes of Health.

## Conflict of Interest

RAT receives grant funding from Beckman Coulter for unrelated work pertaining to artificial intelligence development around emergency department triage. The authors otherwise declare that they have no known competing financial interests or personal relationships that could have appeared to influence the work reported in this paper. PG, DS, AKV, KH, and SW also report receiving support from the Elevance Foundation for Opioid Response Efforts to support a national ED-based quality improvement network directed at opioid use disorder.
