# Supplementary material for: Mapping Emergency Medicine Data to the Observational Medical Outcomes Partnership Common Data Model: A Gap Analysis of the American College of Emergency Physicians Clinical Emergency Data Registry
Source: J Am Coll Emerg Physicians Open. 2025 Jan 10;6(1):100016. doi: 10.1016/j.acepjo.2024.100016 (PMC11853007; doi:10.1016/j.acepjo.2024.100016)
Supplement: Supplementary Appendix 1 [file mmc1.pdf]

Supplemental Table

Supplemental Table 1. Excel file of the comparison matrix mapping fields from CEDR to the OMOP-CDM. To view or download the file please go to https://osf.io/av6mj/?view_only=24e9c4a1c1844268a61a0739f32e25ff
Abbreviations: CEDR, Clinical Emergency Data Registry; Observational Medical Outcomes Partnership Common Data Model, OMOP-CDM

CEDR Table	CEDR Attribute Name	CEDR Description	CEDR DataType	CEDR Sample Data	OMOP Table	OMOP Attribute Name	OMOP User Guide	OMOP ETL Conventions	OMOP DataType	Field Comparison	Comments/Questions
PATIENT	patient_id	An identifier unique to the patient, may be the MRN	nvarchar	12345678	PERSON	person_id	It is assumed that every person with a different unique identifier is in fact a different person and should be treated independently.	Any person linkage that needs to occur to uniquely identify Persons ought to be done prior to writing this table. This identifier can be the original id from the source data provided if it is an integer, otherwise it can be an autogenerated number.	integer	Match and Transform	IC: Could be either person_id or person_source value depending on whether MRN is used. If MRN used, will need to autogenerate a number
PATIENT	patient_mrn	Medical Record Number for the patient	nvarchar	12345678	PERSON	person_source_value	Use this field to link back to persons in the source data. This is typically used for error checking of ETL logic.	Some use cases require the ability to link back to persons in the source data. This field allows for the storing of the person value as it appears in the source. This field is not required but strongly recommended.	varchar(50)	Match	
PATIENT	patient_ssn	Unique Social Security Number for the patient [optional]	nvarchar	000-111-1111						No equivalent in OMOP	
PATIENT	patient_first_name	Patient's First name	nvarchar	John						No equivalent in OMOP	IC: We could place it in the source_value column but it is discouraged, or add a column. https://forums.ohdsi.org/t/patent-name-in-omop-db/18449/4
PATIENT	patient_last_name	Patient's Last name	nvarchar	Bogle						No equivalent in OMOP	IC: We could place it in the source_value column but it is discouraged, or add a column. https://forums.ohdsi.org/t/patent-name-in-omop-db/18449/4
PATIENT	patient_gender	Patient's Gender	nvarchar	M	PERSON	gender_source_value	This field is used to store the biological sex of the person from the source data. It is not intended for use in standard analytics but for reference only.	Put the biological sex of the person as it appears in the source data.	varchar(50)	Match	IC: Need to ensure that it is sex rather than gender
PATIENT	patient_date_of_birth	Patient's Date of birth	date	2000-01-01	PERSON	birth_datetime		This field is not required but highly encouraged. For data sources that provide the precise datetime of birth, that value should be stored in this field. If birth_datetime is not provided in the source, use the following logic to infer the date: If day_of_birth is null and month_of_birth is not null then use the first of the month in that year. If month_of_birth is null or if day_of_birth AND month_of_birth are both null and the person has records during their year of birth then use the date of the earliest record, otherwise use the 15th of June of that year. If time of birth is not given use midnight (00:00:0000).	datetime	Match and Transform	IC: Change from date to datetime assume midnight. Will also need to use this field to engineer birth_day, birth_month, and birth_year
PATIENT	patient_date_of_death	If patient is deceased, the death date	date	NULL	DEATH	death_date	The date the person was deceased.	If the precise date include day or month is not known or not allowed, December is used as the default month, and the last day of the month the default day.	date	Match	
PATIENT	patient_race	Patient's Race description	nvarchar	white	PERSON	race_source_value	This field is used to store the race of the person from the source data. It is not intended for use in standard analytics but for reference only.	Put the race of the person as it appears in the source data.	varchar(50)	Match	
PATIENT	patient_ethnicity	Patient's Ethnicity description	nvarchar	Non-Hispanic	PERSON	ethnicity_source_value	This field is used to store the ethnicity of the person from the source data. It is not intended for use in standard analytics but for reference only.	If the person has an ethnicity other than the OMB standard of "Hispanic" or "Not Hispanic" store that value from the source data here.	varchar(50)	Match	
PATIENT	patient_language	Patient's documented preferred spoken language	nvarchar	English	OBSERVATION	value_as_string	This is the categorical value of the Result of the Observation, if applicable and available.			Match and Transform	IC: We can set observation_source_value to "Language". Similar to this example: https://physionet.org/content/mimic-iv-demo-omop/0.9/1_omop_data_csv/observation.csv
PATIENT	service_location_id	Unique identifier for the encounter place of service	nvarchar	324325352	PERSON	care_site_id		Assign an ID to each combination of a location and nature of the site - the latter could be the Place of Service, name or another characteristic in your source data.	integer	Match	
PATIENT	service_location_name	Text description of the encounter place of service	nvarchar	State Hospital	CARE_SITE	care_site_name	The name of the care_site as it appears in the source data		varchar(255)	Match	
PATIENT	payer_id	Unique identifier number assigned to the insured person's insurance company	nvarchar	01112	PAYER_PLAN_PERIOD	payer_source_value	This is the Payer as it appears in the source data.		varchar(50)	Match	IC: Double check that it is not payer_source_concept_id or plan_source_value or any of the other attributes in the PAYER_PLAN_PERIOD table
ENCOUNTER	patient_id	An identifier unique to the patient	nvarchar	48729374	VISIT_OCCURRENCE	person_id	It is assumed that every person with a different unique identifier is in fact a different person and should be treated independently.	Any person linkage that needs to occur to uniquely identify Persons ought to be done prior to writing this table. This identifier can be the original id from the source data provided if it is an integer, otherwise it can be an autogenerated number.	integer	Match and Transform	IC: Could be either person_id or person_source value depending on whether MRN is used. If MRN used, will need to autogenerate a number
ENCOUNTER	encounter_id	An identifier unique to the encounter	nvarchar	101	VISIT_OCCURRENCE	visit_occurrence_id	Use this to identify unique interactions between a person and the health care system. This identifier links across the other CDM event tables to associate events with a visit.	This should be populated by creating a unique identifier for each unique interaction between a person and the healthcare system where the person receives a medical good or service over a span of time.	integer	Match	
ENCOUNTER	encounter_type_code	CPT code used to indicate the Evaluation and Management (E/M) or Critical Care services coded for the specific encounter. Use additional rows to submit multiple codes for the encounter.	nvarchar	99283	OBSERVATION	observation_source_value	This field houses the verbatim value from the source data representing the Observation that occurred. For example, this could be an ICD10 or Read code.	This code is mapped to a Standard Concept in the Standardized Vocabularies and the original code is stored here for reference.	varchar(50)	Match	IC: Non-standard concept. https://athena.ohdsi.org/search-terms/terms?standardConcept=Standard&standardConcept=Non-standard&vocabulary=CPT4&page=1&pageSize=15&query=99283%C2%A0
ENCOUNTER	encounter_type_code_modifier	One or more CPT code modifiers utilized in addition to the encounter type CPT code	nvarchar	25						No equivalent in OMOP	
ENCOUNTER	encounter_type_description	Text description of the Encounter Type Code	nvarchar	Emergency department visit for the evaluation and management of a patient	OBSERVATION	value_source_value	This field houses the verbatim result value of the Observation from the source data. Do not get confused with the Observation_source_value which captures source value of the observation mapped to observation_concept_id. This field is the observation result value from the source.	If the observation_source_value was a question, for example, or an observation that requires a result then this field is the answer/ result from the source data. Store the verbatim value that represents the result of the observation_source_value.	varchar(50)	Match	
ENCOUNTER	service_provider_npi	The unique NPI for the clinician providing the encounter services	nvarchar	5555555551	PROVIDER	npi	This is the National Provider Number issued to health care providers in the US by the Centers for Medicare and Medicaid Services (CMS).		varchar(20)	Match	
ENCOUNTER	service_provider_first_name	First name of the clinician providing the encounter services	nvarchar	Sam	PROVIDER	provider_name		This field is not necessary as it is not necessary to have the actual identity of the Provider. Rather, the idea is to uniquely and anonymously identify providers of care across the database.	varchar(255)	Match and Transform	IC: Need to combine first_name and last_name
ENCOUNTER	service_provider_last_name	Last name of the clinician providing the encounter services	nvarchar	Jones	PROVIDER	provider_name		This field is not necessary as it is not necessary to have the actual identity of the Provider. Rather, the idea is to uniquely and anonymously identify providers of care across the database.	varchar(255)	Match and Transform	IC: Need to combine first_name and last_name
ENCOUNTER	arrival_date_time	Date and time when the patient arrives in the ED facility [optional]	datetime	2021-01-01 18:35:00	VISIT_OCCURRENCE			If no time is given for the start date of a visit, set it to midnight (00:00:0000).	datetime	Match	
ENCOUNTER	encounter_start_date_time	Date and time when the clinician encounter with the patient begins	datetime	2021-01-01 18:40:00	VISIT_DETAIL	visit_detail_start_datetime		If no time is given for the start date of a visit, set it to midnight (00:00:0000).	datetime	Match	
ENCOUNTER	encounter_end_date_time	Date and time when the clinician encounter with the patient ends	datetime	2021-01-01 22:40:00	VISIT_DETAIL	visit_detail_end_datetime	If a Person is still an inpatient in the hospital at the time of the data extract and does not have a visit_end_datetime, then set the visit_end_datetime to the datetime of the data pull.	If no time is given for the end date of a visit, set it to midnight (00:00:0000).	datetime	Match	
ENCOUNTER	service_location_id	Unique identifier for the encounter place of service	nvarchar	324325352	VISIT_OCCURRENCE	care_site_id		Assign an ID to each combination of a location and nature of the site - the latter could be the Place of Service, name or another characteristic in your source data.	integer	Match	
ENCOUNTER	service_location_name	Text description of the encounter place of service	nvarchar	State Hospital	CARE_SITE	care_site_name	The name of the care_site as it appears in the source data		varchar(255)	Match	
ENCOUNTER	service_location_role_type_code	Code for the type of encounter service location, e.g., Emergency Department (ED), Urgent Care (UC), Fast Track (FTR) [optional]	nvarchar	ED	VISIT_OCCURRENCE	admitted_from_concept_id	Use this field to determine where the patient was admitted from. This concept is part of the visit domain and can indicate if a patient was admitted to the hospital from a long-term care facility, for example.	If available, map the admitted_from_source_value to a standard concept in the visit domain. Accepted Concepts. If a person was admitted from home, set this to 0.	integer	Match and Transform	
ENCOUNTER	service_location_role_type_description	Description for Service Location Role Type [optional]	nvarchar	Emergency Department	VISIT_OCCURRENCE	admitted_from_source_value		This information may be called something different in the source data but the field is meant to contain a value indicating where a person was admitted from. Typically this applies only to visits that have a length of stay, like inpatient visits or long-term care visits.	varchar(50)	Match	
ENCOUNTER	encounter_status	Text description of the disposition of the patient from the ED	nvarchar	Discharge	VISIT_DETAIL	visit_detail_source_value	This field houses the verbatim value from the source data representing the kind of visit detail that took place (inpatient, outpatient, emergency, etc.)	If there is information about the kind of visit detail in the source data that value should be stored here. If a visit is an amalgamation of visits from the source then use a hierarchy to choose the VISIT_DETAIL_SOURCE_VALUE, such as IP -> ER-> OP. This should line up with the logic chosen to determine how visits are created.		Match and Transform	
ENCOUNTER	discharge_disposition_description	Text description of the final disposition of the patient	nvarchar	Discharge to home	VISIT_OCCURRENCE	discharged_to_source_value		This information may be called something different in the source data but the field is meant to contain a value indicating where a person was discharged to after a visit, as in they went home or were moved to long-term care. Typically this applies only to visits that have a length of stay of a day or more.	varchar(50)	Match	
ENCOUNTER	discharge_disposition_code	Standard code representing the final disposition status of the patient	nvarchar	01	VISIT_OCCURRENCE	discharged_to_concept_id	Use this field to determine where the patient was discharged to after a visit. This concept is part of the visit domain and can indicate if a patient was transferred to another hospital or sent to a long-term care facility, for example. It is assumed that a person is discharged to home therefore there is not a standard concept id for "home". Use concept id = 0 when a person is discharged to home.	If available, map the discharged_to_source_value to a standard concept in the visit domain. .	integer	Match and Transform	IC: Need to check whether the CEDR matches to an accepted concept. https://athena.ohdsi.org/search-terms/terms?domain=Visit&standardConcept=Standard&page=1&pageSize=15&query=
ENCOUNTER	discharge_date_time	Date and time the patient was discharged from the encounter	datetime	2021-01-01 22:40:00	VISIT_OCCURRENCE	visit_end_datetime	If a Person is still an inpatient in the hospital at the time of the data extract and does not have a visit_end_datetime, then set the visit_end_datetime to the datetime of the data pull.	If no time is given for the end date of a visit, set it to midnight (00:00:0000).	datetime	Match	
ENCOUNTER	departure_date_time	Date and time the patient leaves ED facility [optional]	datetime	2021-01-01 22:45:00	VISIT_OCCURRENCE	visit_end_datetime	If a Person is still an inpatient in the hospital at the time of the data extract and does not have a visit_end_datetime, then set the visit_end_datetime to the datetime of the data pull.	If no time is given for the end date of a visit, set it to midnight (00:00:0000).	datetime	Match	
ENCOUNTER DIAGNOSIS	patient_id	An identifier unique to the patient	nvarchar	18394792	CONDITION_OCCURRENCE	person_id	The PERSON_ID of the PERSON for whom the condition is recorded.		integer	Match and Transform	IC: Could be either person_id or person_source value depending on whether MRN is used. If MRN used, will need to autogenerate a number
ENCOUNTER DIAGNOSIS	encounter_id	An identifier unique to the encounter	nvarchar	105	CONDITION_OCCURRENCE	visit_occurrence_id	The visit during which the condition occurred.	Depending on the structure of the source data, this may have to be determined based on dates. If a CONDITION_START_DATE occurs within the start and end date of a Visit it is a valid ETL choice to choose the VISIT_OCCURRENCE_ID from the Visit that subsumes it, even if not explicitly stated in the data. While not required, an attempt should be made to locate the VISIT_OCCURRENCE_ID of the CONDITION_OCCURRENCE record.	integer	Match	
ENCOUNTER DIAGNOSIS	encounter_diagnosis_code	Include the primary and additional diagnosis codes related to the encounter, represented as ICD10, SNOMED, or CT codes	nvarchar	S09.90	CONDITION_OCCURRENCE	condition_source_value	This field houses the verbatim value from the source data representing the condition that occurred. For example, this could be an ICD10 or Read code.	This code is mapped to a Standard Condition Concept in the Standardized Vocabularies and the original code is stored here for reference.	varchar(50)	Match	
ENCOUNTER DIAGNOSIS	encounter_diagnosis_code_description	Text description of the encounter diagnosis codes	nvarchar	Head Injury	CONCEPT	concept_name	An unambiguous, meaningful and descriptive name for the Concept.		varchar(255)	Match	
ENCOUNTER DIAGNOSIS	diagnosis_code_set	This value indicates the type of the encounter diagnosis code set	nvarchar	ICD10	VOCABULARY	vocabulary_id	A unique identifier for each Vocabulary, such as ICD9CM, SNOMED, Visit.		varchar(20)	Match	IC: Should be vocabulary_id based on this: https://www.ohdsi.org/web/wiki/doku.php?id=documentation:cdm:vocabulary
ENCOUNTER DIAGNOSIS	encounter_documentation_date_time	Date and time that the encounter diagnosis was documented	datetime	2021-01-01 18:45:00	CONDITION_OCCURRENCE	condition_start_datetime	If a source does not specify datetime the convention is to set the time to midnight (00:00:0000)	If a source does not specify datetime the convention is to set the time to midnight (00:00:0000)	datetime	Match	
ENCOUNTER DIAGNOSIS	onset_date_time	Date and time when the diagnosis was reported to begin or was first observed	datetime	2021-01-01 18:40:00	OBSERVATION	observation_datetime, concept_id		If no time is given set to midnight (00:00:00).	datetime	Match and Transform	IC:Need to create a Concept_id for "date of onset" (4181873 Link between the diagnosis record in CONDITION_OCCURENCE table and the date of onset record in OBSERVATION table should be established via FACT_RELATIONSHIP table See more here: https://forums.ohdsi.org/t/how-to-store-date-of-onset-and-date-of-diagnosis-for-a-disease/9460
ENCOUNTER DIAGNOSIS	resolution_date_time	Date and time when the diagnosis was resolved	datetime	2021-01-01 22:40:00	CONDITION_OCCURRENCE	condition_end_datetime		If a source does not specify datetime the convention is to set the time to midnight (00:00:0000)	datetime	Match	
ENCOUNTER DIAGNOSIS	service_provider_npi	The unique NPI for the clinician providing the encounter services	nvarchar	5555555555	PROVIDER	npi	This is the National Provider Number issued to health care providers in the US by the Centers for Medicare and Medicaid Services (CMS).		varchar(20)	Match	
ENCOUNTER DIAGNOSIS	service_provider_first_name	First name of the clinician providing the billed encounter services	nvarchar	Linda	PROVIDER	provider_name		This field is not necessary as it is not necessary to have the actual identity of the Provider. Rather, the idea is to uniquely and anonymously identify providers of care across the database.	varchar(255)	Match and Transform	IC: Need to combine first_name and last_name
ENCOUNTER DIAGNOSIS	service_provider_last_name	Last name of the clinician providing the billed encounter services	nvarchar	Smith	PROVIDER	provider_name		This field is not necessary as it is not necessary to have the actual identity of the Provider. Rather, the idea is to uniquely and anonymously identify providers of care across the database.	varchar(255)	Match and Transform	IC: Need to combine first_name and last_name
ENCOUNTER DIAGNOSIS	service_provider_role_code	Codes that classify providers according to the role they play in the healthcare setting [optional]	nvarchar	N						No equivalent in OMOP	
ENCOUNTER DIAGNOSIS	service_provider_role_description	Text description for Service Provider Role Code [optional]	nvarchar	Nurse	PROVIDER	specialty_source_value	This is the kind of provider or specialty as it appears in the source data. This includes physician specialties such as internal medicine, emergency medicine, etc. and allied health professionals such as nurses, midwives, and pharmacists.	Put the kind of provider as it appears in the source data. This field is up to the discretion of the ETL-er as to whether this should be the coded value from the source or the text description of the lookup value.	varchar(50)	Match	
ENCOUNTER DIAGNOSIS	primary_diagnosis	Field to state if this is the primary Encounter Diagnosis	nvarchar	No	CONDITION_OCCURRENCE	condition_status_concept_id	This concept represents the point during the visit the diagnosis was given (admitting diagnosis, final diagnosis), whether the diagnosis was determined due to laboratory findings, if the diagnosis was exclusionary, or if it was a preliminary diagnosis, among others.	Choose the Concept in the Condition Status domain that best represents the point during the visit when the diagnosis was given. These can include admitting diagnosis, principal diagnosis, and secondary diagnosis. Accepted Concepts.	integer	Match and Transform	IC: https://forums.ohdsi.org/t/primary-diagnosis-as-condition-status/8062
ENCOUNTER DIAGNOSIS	service_location_id	Unique identifier for the encounter place of service	nvarchar	848383005	VISIT_OCCURRENCE	care_site_id		Assign an ID to each combination of a location and nature of the site - the latter could be the Place of Service, name or another characteristic in your source data.	integer	Match	
ENCOUNTER DIAGNOSIS	service_location_name	Text description of the encounter place of service	nvarchar	Smile Hospital	CARE_SITE	care_site_name	The name of the care_site as it appears in the source data		varchar(255)	Match	
ENCOUNTER PATIENT COMPLAINT	encounter_id	An identifier unique to the encounter	nvarchar	105	CONDITION_OCCURRENCE	visit_occurrence_id	The visit during which the condition occurred.	Depending on the structure of the source data, this may have to be determined based on dates. If a CONDITION_START_DATE occurs within the start and end date of a Visit it is a valid ETL choice to choose the VISIT_OCCURRENCE_ID from the Visit that subsumes it, even if not explicitly stated in the data. While not required, an attempt should be made to locate the VISIT_OCCURRENCE_ID of the CONDITION_OCCURRENCE record.	integer	Match	
ENCOUNTER PATIENT COMPLAINT	patient_id	An identifier unique to the patient	nvarchar	12374389	CONDITION_OCCURRENCE	person_id	The PERSON_ID of the PERSON for whom the condition is recorded.		integer	Match	
ENCOUNTER PATIENT COMPLAINT	patient_complaint_description	Text description of complaints captured from patient encounter. Also known as reason for visit.	nvarchar(MAX)	Superficial cut needing stitches	CONDITION_OCCURRENCE	condition_source_value	This field houses the verbatim value from the source data representing the condition that occurred. For example, this could be an ICD10 or Read code.	This code is mapped to a Standard Condition Concept in the Standardized Vocabularies and the original code is stored here for reference.	varchar(50)	Match and Transform	IC: Chief complaint should go in the CONDITION_OCCURENCE table. Use condition_type_concept_id to specify chief complaint? See more here: https://forums.ohdsi.org/t/proper-table-for-chief-complaint-reason-for-visit/12939
ENCOUNTER PATIENT COMPLAINT	patient_primary_complaint	Is the documented patient complaint the primary complaint of the patient from the encounter	nvarchar	Yes	CONDITION_OCCURRENCE	condition_status_source_value	This field houses the verbatim value from the source data representing the condition status.	This information may be called something different in the source data but the field is meant to contain a value indicating when and how a diagnosis was given to a patient. This source value is mapped to a standard concept which is stored in the CONDITION_STATUS_CONCEPT_ID field.	varchar(50)	Match	
ENCOUNTER PATIENT COMPLAINT	patient_complaint_documentation_date_time	The date and time that the patient complaint was documented in the system	datetime	2021-01-01 16:40:00	CONDITION_OCCURRENCE	condition_start_datetime		If a source does not specify datetime the convention is to set the time to midnight (00:00:0000)	datetime	Match	
ENCOUNTER PATIENT COMPLAINT	service_location_id	Unique identifier for the encounter place of service	nvarchar	848383005	VISIT_OCCURRENCE	care_site_id		Assign an ID to each combination of a location and nature of the site - the latter could be the Place of Service, name or another characteristic in your source data.	integer	Match	
ENCOUNTER PATIENT COMPLAINT	service_location_name	Text description of the encounter place of service	nvarchar	Smile Hospital	CARE_SITE	care_site_name	The name of the care_site as it appears in the source data		varchar(255)	Match	
RESULT OBSERVATION	patient_id	An identifier unique to the patient	nvarchar	18372932	MEASUREMENT	person_id	The PERSON_ID of the Person for whom the Measurement is recorded. This may be a system generated code.		integer	Match	
RESULT OBSERVATION	encounter_id	An identifier unique to the encounter	nvarchar	105	MEASUREMENT	visit_occurrence_id	The visit during which the Measurement occurred.	Depending on the structure of the source data, this may have to be determined based on dates. If a MEASUREMENT_DATE occurs within the start and end date of a Visit it is a valid ETL choice to choose the VISIT_OCCURRENCE_ID from the visit that subsumes it, even if not explicitly stated in the data. While not required, an attempt should be made to locate the VISIT_OCCURRENCE_ID of the measurement record. If a measurement is related to a visit explicitly in the source data, it is possible that the result date of the Measurement falls outside of the bounds of the Visit dates.	integer	Match	
RESULT OBSERVATION	observation_code	Standard codes that describe the observations of the results of laboratory orders, imaging procedures, and other studies.	nvarchar	10466-1	MEASUREMENT	measurement_source_value	This field houses the verbatim value from the source data representing the Measurement that occurred. For example, this could be an ICD10 or Read code.	This code is mapped to a Standard Measurement Concept in the Standardized Vocabularies and the original code is stored here for reference.	varchar(50)	Match	
RESULT OBSERVATION	observation_description	Text description for Observation Code	nvarchar	Anion gap 3 in Serum or Plasma	CONCEPT	concept_name	An unambiguous, meaningful and descriptive name for the Concept.		varchar(255)	Match	
RESULT OBSERVATION	result_code_set	Code set which is used in column Observation Code	nvarchar	LOINC	VOCABULARY	vocabulary_id	A unique identifier for each Vocabulary, such as ICD9CM, SNOMED, Visit.		varchar(20)	Match	IC: Should be vocabulary_id based on this: https://www.ohdsi.org/web/wiki/doku.php?id=documentation:cdm:vocabulary
RESULT OBSERVATION	result_date_time	Date and time of the result/observation	datetime	2019-01-01 18:35:00	MEASUREMENT	measurement_datetime		This is not required, though it is in v6. If a source does not specify datetime the convention is to set the time to midnight (00:00:0000)	datetime	Match	
RESULT OBSERVATION	result_value	The documented value/result	nvarchar	4	MEASUREMENT	value_source_value	This field houses the verbatim result value of the Measurement from the source data .	If both a continuous and categorical result are given in the source data such that both VALUE_AS_NUMBER and VALUE_AS_CONCEPT_ID are both included, store the verbatim value that was mapped to VALUE_AS_CONCEPT_ID here.	varchar(50)	Match and Transform	IC: Remember to convert Blood Pressure.
RESULT OBSERVATION	result_flag	Standard observation interpretation code of the result	nvarchar	N						No equivalent in OMOP	
RESULT OBSERVATION	result_flag_description	Text description of the Result Flag	nvarchar	Normal	MEASUREMENT	value_as_concept_id	If the raw data gives a categorial result for measurements those values are captured and mapped to standard concepts in the 'Meas Value' domain.	If the raw data provides categorial results as well as continuous results for measurements, it is a valid ETL choice to preserve both values. The continuous value should go in the VALUE_AS_NUMBER field and the categorical value should be mapped to a standard concept in the 'Meas Value' domain and put in the VALUE_AS_CONCEPT_ID field. This is also the destination for the 'Maps to value' relationship.	integer	Match and Transform	IC: https://forums.ohdsi.org/t/measurement-table-are-value-as-concept-id-and-value-as-number-mutually-exclusive/10975
RESULT OBSERVATION	normal_range_low	Lower limit of normal result range	nvarchar	3	MEASUREMENT	range_low	Ranges have the same unit as the VALUE_AS_NUMBER. These ranges are provided by the source and should remain NULL if not given.	If reference ranges for upper and lower limit of normal as provided (typically by a laboratory) these are stored in the RANGE_HIGH and RANGE_LOW fields. This should be set to NULL if not provided.	float	Match	
RESULT OBSERVATION	normal_range_high	Upper limit of normal result range	nvarchar	10	MEASUREMENT	range_high	Ranges have the same unit as the VALUE_AS_NUMBER. These ranges are provided by the source and should remain NULL if not given.	If reference ranges for upper and lower limit of normal as provided (typically by a laboratory) these are stored in the RANGE_HIGH and RANGE_LOW fields. This should be set to NULL if not provided.	float	Match	
RESULT OBSERVATION	result_unit	The result unit	nvarchar	mEq/L	MEASUREMENT	unit_source_value	This field houses the verbatim value from the source data representing the unit of the Measurement that occurred.	This code is mapped to a Standard Condition Concept in the Standardized Vocabularies and the original code is stored here for reference.	varchar(50)	Match	
RESULT OBSERVATION	order_id	Unique identifier identifying the patient's order for which the result was recorded. Used to link the result in Result Observation table to the Order in the Orders table.	nvarchar	2432	MEASUREMENT	measurement_id	The unique key given to a Measurement record for a Person. Refer to the ETL for how duplicate Measurements during the same Visit were handled.	Each instance of a measurement present in the source data should be assigned this unique key. In some cases, a person can have multiple records of the same measurement within the same visit. It is valid to keep these duplicates and assign them individual, unique, MEASUREMENT_IDs, though it is up to the ETL how they should be handled.	integer	Match	
RESULT OBSERVATION	result_order_description	Text description of the result order	nvarchar	Basic metabolic panel	CONCEPT	concept_name	An unambiguous, meaningful and descriptive name for the Concept.		varchar(255)	Match and Transform	IC: May need to create a FACT RELATIONSHIP and have a parent child relationship with specific lab tests
RESULT OBSERVATION	service_provider_npi	The unique NPI for the ordering clinician	nvarchar	5555555555	PROVIDER	npi	This is the National Provider Number issued to health care providers in the US by the Centers for Medicare and Medicaid Services (CMS).		varchar(20)	Match	
RESULT OBSERVATION	service_provider_first_name	First name of the ordering clinician	nvarchar	Linda	PROVIDER	provider_name		This field is not necessary as it is not necessary to have the actual identity of the Provider. Rather, the idea is to uniquely and anonymously identify providers of care across the database.	varchar(255)	Match and Transform	IC: Need to combine first_name and last_name
RESULT OBSERVATION	service_provider_last_name	Last name of the ordering clinician	nvarchar	Smith	PROVIDER	provider_name		This field is not necessary as it is not necessary to have the actual identity of the Provider. Rather, the idea is to uniquely and anonymously identify providers of care across the database.	varchar(255)	Match and Transform	IC: Need to combine first_name and last_name
RESULT OBSERVATION	service_location_id	Unique identifier for the encounter place of service	nvarchar	848383005	VISIT_OCCURRENCE	care_site_id		Assign an ID to each combination of a location and nature of the site - the latter could be the Place of Service, name or another characteristic in your source data.	integer	Match	
RESULT OBSERVATION	service_location_name	Text description of the encounter place of service	nvarchar	Smile Hospital	CARE_SITE	care_site_name	The name of the care_site as it appears in the source data		varchar(255)	Match	
ENCOUNTER PROCEDURE	patient_id	An identifier unique to the patient	nvarchar	121212	PROCEDURE_OCCURRENCE	person_id	The PERSON_ID of the PERSON for whom the procedure is recorded. This may be a system generated code.		integer	Match	
ENCOUNTER PROCEDURE	encounter_id	An identifier unique to the encounter	nvarchar	105	PROCEDURE_OCCURRENCE	visit_occurrence_id	The visit during which the procedure occurred.	Depending on the structure of the source data, this may have to be determined based on dates. If a PROCEDURE_DATE occurs within the start and end date of a Visit it is a valid ETL choice to choose the VISIT_OCCURRENCE_ID from the Visit that subsumes it, even if not explicitly stated in the data. While not required, an attempt should be made to locate the VISIT_OCCURRENCE_ID of the PROCEDURE_OCCURRENCE record.	integer	Match	
ENCOUNTER PROCEDURE	service_provider_npi	The unique NPI for the clinician providing the encounter services	nvarchar	5555555555	PROVIDER	npi	This is the National Provider Number issued to health care providers in the US by the Centers for Medicare and Medicaid Services (CMS).		varchar(20)	Match	
ENCOUNTER PROCEDURE	service_provider_first_name	First name of the clinician providing the billed encounter services	nvarchar	Linda	PROVIDER	provider_name		This field is not necessary as it is not necessary to have the actual identity of the Provider. Rather, the idea is to uniquely and anonymously identify providers of care across the database.	varchar(255)	Match and Transform	IC: Need to combine first_name and last_name
ENCOUNTER PROCEDURE	service_provider_last_name	Last name of the clinician providing the billed encounter services	nvarchar	Smith	PROVIDER	provider_name		This field is not necessary as it is not necessary to have the actual identity of the Provider. Rather, the idea is to uniquely and anonymously identify providers of care across the database.	varchar(255)	Match and Transform	IC: Need to combine first_name and last_name
ENCOUNTER PROCEDURE	procedure_code	One to many standard codes that represent a procedure, surgery, or performance measure performed during the encounter. Code types may include ICD10 PCS, CPT, SNOMED CT, CPT II, HCPCS II ICD10 PCS, CPT, SNOMED CT, CPT II, HCPCS II.	nvarchar	31500	PROCEDURE_OCCURRENCE	procedure_source_value	This field houses the verbatim value from the source data representing the procedure that occurred. For example, this could be an CPT4 or OPCS4 code.	Use this value to look up the source concept id and then map the source concept id to a standard concept id.	varchar(50)	Match	
ENCOUNTER PROCEDURE	procedure_code_modifier	Include any modifiers to the Procedure Code	nvarchar	51	PROCEDURE_OCCURRENCE	modifier_source_value		The original modifier code from the source is stored here for reference.	varchar(50)	Match	
ENCOUNTER PROCEDURE	procedure_description	Standard description of the procedure, surgery, or performance measure codified by the Procedure Code	nvarchar	Endotracheal Intubation	CONCEPT	concept_name	An unambiguous, meaningful and descriptive name for the Concept.		varchar(255)	Match	
ENCOUNTER PROCEDURE	procedure_code_set	Code set for the Procedure Code including ICD10 PCS, CPT, SNOMED CT, CPT II, HCPCS II	nvarchar	CPT	VOCABULARY	vocabulary_id	A unique identifier for each Vocabulary, such as ICD9CM, SNOMED, Visit.		varchar(20)	Match	
ENCOUNTER PROCEDURE	procedure_date_time	Date and time of the procedure	datetime	2022-01-01 18:35:00	PROCEDURE_OCCURRENCE	procedure_datetime		If the procedure has a start time in the native date, use this field to house that information. This will be renamed in a future version to PROCEDURE_START_DATETIME.	datetime	Match	
ENCOUNTER PROCEDURE	service_location_id	Unique identifier for the encounter place of service	nvarchar	848383005	VISIT_OCCURRENCE	care_site_id		Assign an ID to each combination of a location and nature of the site - the latter could be the Place of Service, name or another characteristic in your source data.	integer	Match	
ENCOUNTER PROCEDURE	service_location_name	Text description of the encounter place of service	nvarchar	Smile Hospital	CARE_SITE	care_site_name	The name of the care_site as it appears in the source data		varchar(255)	Match	
VITAL SIGNS	patient_id	An identifier unique to the patient	nvarchar	121212	MEASUREMENT	person_id	The PERSON_ID of the Person for whom the Measurement is recorded. This may be a system generated code.		integer	Match	
VITAL SIGNS	encounter_id	An identifier unique to the encounter	nvarchar	101	MEASUREMENT	visit_occurrence_id	The visit during which the Measurement occurred.	Depending on the structure of the source data, this may have to be determined based on dates. If a MEASUREMENT_DATE occurs within the start and end date of a Visit it is a valid ETL choice to choose the VISIT_OCCURRENCE_ID from the visit that subsumes it, even if not explicitly stated in the data. While not required, an attempt should be made to locate the VISIT_OCCURRENCE_ID of the measurement record. If a measurement is related to a visit explicitly in the source data, it is possible that the result date of the Measurement falls outside of the bounds of the Visit dates.	integer	Match	
VITAL SIGNS	vital_sign_code	Standard vital sign code, preferably LOINC codes	nvarchar	8302-2	MEASUREMENT	measurement_source_value	This field houses the verbatim value from the source data representing the Measurement that occurred. For example, this could be an ICD10 or Read code.	This code is mapped to a Standard Measurement Concept in the Standardized Vocabularies and the original code is stored here for reference.	varchar(50)	Match	
VITAL SIGNS	vital_sign_description	Text description of the vital sign code	nvarchar	Body height	CONCEPT	concept_name	An unambiguous, meaningful and descriptive name for the Concept.		varchar(255)	Match	
VITAL SIGNS	vital_sign_value	Actual value of the vital sign	nvarchar	177	MEASUREMENT	value_source_value	This field houses the verbatim result value of the Measurement from the source data .	If both a continuous and categorical result are given in the source data such that both VALUE_AS_NUMBER and VALUE_AS_CONCEPT_ID are both included, store the verbatim value that was mapped to VALUE_AS_CONCEPT_ID here.	varchar(50)	Match	
VITAL SIGNS	vital_sign_unit	This is the unit of the vital sign (e.g., "cm", %)	nvarchar	cm	MEASUREMENT	unit_source_value	This field houses the verbatim value from the source data representing the unit of the Measurement that occurred.	This code is mapped to a Standard Condition Concept in the Standardized Vocabularies and the original code is stored here for reference.	varchar(50)	Match	
VITAL SIGNS	vital_sign_documentation_date	Date and time the vital sign was documented in the system	datetime	2022-01-05 18:35:00	MEASUREMENT	measurement_datetime		This is not required, though it is in v6. If a source does not specify datetime the convention is to set the time to midnight (00:00:0000)	datetime	Match	
VITAL SIGNS	service_location_id	Unique identifier for the encounter place of service	nvarchar	848383005	VISIT_OCCURRENCE	care_site_id		Assign an ID to each combination of a location and nature of the site - the latter could be the Place of Service, name or another characteristic in your source data.	integer	Match	
VITAL SIGNS	service_location_name	Text description of the encounter place of service	nvarchar	Smile Hospital	CARE_SITE	care_site_name	The name of the care_site as it appears in the source data		varchar(255)	Match	
PATIENT PROBLEM	patient_id	An identifier unique to the patient	nvarchar	121212	CONDITION_OCCURRENCE, OBSERVATION	person_id	The PERSON_ID of the PERSON for whom the condition is recorded.		integer	Match and Transform	IC: We will need to include historical diagnoses in the OBSERVATION table and current diagnoses in the CONDITIONS table. https://forums.ohdsi.org/t/how-to-capture-patient-history-in-cdmv4/558/2
PATIENT PROBLEM	encounter_id	An identifier unique to the encounter	nvarchar	101	CONDITION_OCCURRENCE, OBSERVATION	visit_occurrence_id	The visit during which the condition occurred.	Depending on the structure of the source data, this may have to be determined based on dates. If a CONDITION_START_DATE occurs within the start and end date of a Visit it is a valid ETL choice to choose the VISIT_OCCURRENCE_ID from the Visit that subsumes it, even if not explicitly stated in the data. While not required, an attempt should be made to locate the VISIT_OCCURRENCE_ID of the CONDITION_OCCURRENCE record.	integer	Match and Transform	IC: We will need to include historical diagnoses in the OBSERVATION table and current diagnoses in the CONDITIONS table. https://forums.ohdsi.org/t/how-to-capture-patient-history-in-cdmv4/558/2
PATIENT PROBLEM	problem_code	Includes data elements representing active and resolved illnesses, injuries, and other key health issues.	nvarchar	R07.11	CONDITION_OCCURRENCE, OBSERVATION	condition_source_value, observation_source_value	This field houses the verbatim value from the source data representing the condition that occurred. For example, this could be an ICD10 or Read code.	This code is mapped to a Standard Condition Concept in the Standardized Vocabularies and the original code is stored here for reference.	varchar(50)	Match and Transform	IC: We will need to include historical diagnoses in the OBSERVATION table and current diagnoses in the CONDITIONS table. https://forums.ohdsi.org/t/how-to-capture-patient-history-in-cdmv4/558/2
PATIENT PROBLEM	problem_description	Text description of the documented problem code	nvarchar	Stroke	CONCEPT	concept_name	An unambiguous, meaningful and descriptive name for the Concept.		varchar(255)	Match	
PATIENT PROBLEM	problem_code_set	Code set used to document the problem code	nvarchar	ICD10	VOCABULARY	vocabulary_id	A unique identifier for each Vocabulary, such as ICD9CM, SNOMED, Visit.		varchar(20)	Match	
PATIENT PROBLEM	problem_documentation_date_time	The date and time that the problem was documented	datetime	2019-12-23 16:35:00	CONDITION_OCCURENCE	condition_start_datetime		If a source does not specify datetime the convention is to set the time to midnight (00:00:0000)	datetime	Match	
PATIENT PROBLEM	problem_onset_date	The date that the patient notes the problem started	date	2019-12-22	OBSERVATION	obvservation_date	The date of the Observation. Depending on what the Observation represents this could be the date of a lab test, the date of a survey, or the date a patient's family history was taken.	For some observations the ETL may need to make a choice as to which date to choose.	date	Match and Transform	IC: Need to create a Concept_id for "date of onset" (4181873 Link between the diagnosis record in CONDITION_OCCURENCE table and the date of onset record in OBSERVATION table should be established via FACT_RELATIONSHIP table See more here: https://forums.ohdsi.org/t/how-to-store-date-of-onset-and-date-of-diagnosis-for-a-disease/9460
PATIENT PROBLEM	problem_resolution_date	The date that the patient notes the problem ended	date	2019-12-23	CONDITION_OCCURRENCE	condition_end_date	Use this date to determine the end date of the condition	Most often data sources do not have the idea of a start date for a condition. Rather, if a source only has one date associated with a condition record it is acceptable to use that date for both the CONDITION_START_DATE and the CONDITION_END_DATE.	date	Match	
PATIENT PROBLEM	service_location_id	Unique identifier for the encounter place of service	nvarchar	848383005	VISIT_OCCURRENCE	care_site_id		Assign an ID to each combination of a location and nature of the site - the latter could be the Place of Service, name or another characteristic in your source data.	integer	Match	
PATIENT PROBLEM	service_location_name	Text description of the encounter place of service	nvarchar	Smile Hospital	CARE_SITE	care_site_name	The name of the care_site as it appears in the source data		varchar(255)	Match	
ORDERS	patient_id	An identifier unique to the patient	nvarchar	121212	MEASUREMENT	person_id	The PERSON_ID of the Person for whom the Measurement is recorded. This may be a system generated code.		integer	Match	
ORDERS	order_id	An identifier unique to the patient's order. Used to link the result in Result Observation table to the Order in the Orders table.	nvarchar	100	MEASUREMENT	measurement_id	The unique key given to a Measurement record for a Person. Refer to the ETL for how duplicate Measurements during the same Visit were handled.	Each instance of a measurement present in the source data should be assigned this unique key. In some cases, a person can have multiple records of the same measurement within the same visit. It is valid to keep these duplicates and assign them individual, unique, MEASUREMENT_IDs, though it is up to the ETL how they should be handled.	integer	Match	
ORDERS	encounter_id	Unique identification for an Encounter	nvarchar	101	MEASUREMENT	visit_occurrence_id	The visit during which the Measurement occurred.	Depending on the structure of the source data, this may have to be determined based on dates. If a MEASUREMENT_DATE occurs within the start and end date of a Visit it is a valid ETL choice to choose the VISIT_OCCURRENCE_ID from the visit that subsumes it, even if not explicitly stated in the data. While not required, an attempt should be made to locate the VISIT_OCCURRENCE_ID of the measurement record. If a measurement is related to a visit explicitly in the source data, it is possible that the result date of the Measurement falls outside of the bounds of the Visit dates.	integer	Match	
ORDERS	order_code	Standard order, i.e., LOINC code for the order	nvarchar	115188	MEASUREMENT	measurement_source_value	This field houses the verbatim value from the source data representing the Measurement that occurred. For example, this could be an ICD10 or Read code.	This code is mapped to a Standard Measurement Concept in the Standardized Vocabularies and the original code is stored here for reference.	varchar(50)	Match	
ORDERS	order_description	Standard text description of the coded order	nvarchar	D dimer	CONCEPT	concept_name	An unambiguous, meaningful and descriptive name for the Concept.		varchar(255)	Match	
ORDERS	order_code_set	Standard code set for the coded order e.g. LOINC, CPT	nvarchar	LOINC	VOCABULARY	vocabulary_id	A unique identifier for each Vocabulary, such as ICD9CM, SNOMED, Visit.		varchar(20)	Match	IC: Should be vocabulary_id based on this: https://www.ohdsi.org/web/wiki/doku.php?id=documentation:cdm:vocabulary
ORDERS	order_date_time	Date and time that the order was placed	datetime	2019-11-14 16:20:00	MEASUREMENT	measurement_datetime		This is not required, though it is in v6. If a source does not specify datetime the convention is to set the time to midnight (00:00:0000)	datetime	Match	IC: Use the documentation datetime or order placed datetime, whichever has the least missing
ORDERS	order_documentation_date_time	Date and time that the order was documented	datetime	2019-11-14 16:20:00	MEASUREMENT	measurement_datetime		This is not required, though it is in v6. If a source does not specify datetime the convention is to set the time to midnight (00:00:0000)	datetime	Match	IC: Use the documentation datetime or order placed datetime, whichever has the least missing
ORDERS	order_status	Indicates the status of the ordered service or procedure	nvarchar	Completed, Cancelled						No equivalent in OMOP	Only completed orders should be placed. https://forums.ohdsi.org/t/measurement/7988, https://forums.ohdsi.org/t/order-request-attributes/16263
ORDERS	service_provider_npi	The unique NPI for the ordering clinician	nvarchar	5555555551	PROVIDER	npi	This is the National Provider Number issued to health care providers in the US by the Centers for Medicare and Medicaid Services (CMS).		varchar(20)	Match	
ORDERS	service_provider_first_name	First name of the ordering clinician	nvarchar	Mary	PROVIDER	provider_name		This field is not necessary as it is not necessary to have the actual identity of the Provider. Rather, the idea is to uniquely and anonymously identify providers of care across the database.	varchar(255)	Match and Transform	IC: Need to combine first_name and last_name
ORDERS	service_provider_last_name	Last name of the ordering clinician	nvarchar	Smith	PROVIDER	provider_name		This field is not necessary as it is not necessary to have the actual identity of the Provider. Rather, the idea is to uniquely and anonymously identify providers of care across the database.	varchar(255)	Match and Transform	IC: Need to combine first_name and last_name
ORDERS	service_location_id	Unique identifier for the encounter place of service	nvarchar	848383005	VISIT_OCCURRENCE	care_site_id		Assign an ID to each combination of a location and nature of the site - the latter could be the Place of Service, name or another characteristic in your source data.	integer	Match	
ORDERS	service_location_name	Text description of the encounter place of service	nvarchar	Smile Hospital	CARE_SITE	care_site_name	The name of the care_site as it appears in the source data		varchar(255)	Match	
PATIENT NOTES	patient_id	An identifier unique to the patient	nvarchar	121212	NOTE	person_id			integer	Match	
PATIENT NOTES	encounter_id	An identifier unique to the encounter	nvarchar	101	NOTE	visit_occurrence_id	The Visit during which the note was written.		integer	Match	
PATIENT NOTES	patient_notes_documentation_date_time	Date and time of the patient note documentation	datetime	2019-11-18 04:20:00	NOTE	note_datetime		If time is not given set the time to midnight.	datetime	Match	
PATIENT NOTES	note_type	Type of patient note	nvarchar	Procedure Notes	NOTE	note_source_value		The source value mapped to the NOTE_CLASS_CONCEPT_ID.	varchar(50)	Match and Transform	IC: Would need to create a note_type_concept_id also. https://athena.ohdsi.org/search-terms/terms?standardConcept=Standard&conceptClass=Doc+Kind&conceptClass=Doc+Role&conceptClass=Doc+Setting&conceptClass=Doc+Subject+Matter&conceptClass=Doc+Type+of+Service&domain=Meas+Value&page=1&pageSize=15&query=procedure+&boosts
PATIENT NOTES	patient_notes	This is a free text field for the notes	nvarchar(MAX)	Simple repair of 2cm laceration	NOTE	note_title, note_text	The title of the note, The content of the note.		varchar(250), varchar(MAX)	Match and Transform	IC: Would need to extract the title from the note to create 2 different variables. We would also need to check to make sure patient_notes1 is not NULL
PATIENT NOTES	patient_notes1	This is an additional field for free text notes if the length of the patient notes exceeds 8000 characters. The remainder can overflow into this field	nvarchar(MAX)		NOTE	note_title, note_text	The title of the note, The content of the note.		varchar(250), varchar(MAX)	Match and Transform	IC: Would need to extract the title from the note to create 2 different variables. We would also need to check to make sure patient_notes1 is not NULL
PATIENT NOTES	service_provider_npi	The unique NPI for the clinician providing the encounter services. If the note recorder does not have an NPI, leave this column blank.	nvarchar	5555555551	PROVIDER	npi	This is the National Provider Number issued to health care providers in the US by the Centers for Medicare and Medicaid Services (CMS).		varchar(20)	Match	
PATIENT NOTES	service_provider_first_name	First name of the clinician providing the billed encounter services	nvarchar	Mary	PROVIDER	provider_name		This field is not necessary as it is not necessary to have the actual identity of the Provider. Rather, the idea is to uniquely and anonymously identify providers of care across the database.	varchar(255)	Match and Transform	IC: Need to combine first_name and last_name
PATIENT NOTES	service_provider_last_name	Last name of the clinician providing the billed encounter services	nvarchar	Smith	PROVIDER	provider_name		This field is not necessary as it is not necessary to have the actual identity of the Provider. Rather, the idea is to uniquely and anonymously identify providers of care across the database.	varchar(255)	Match and Transform	IC: Need to combine first_name and last_name
PATIENT NOTES	service_location_id	Unique identifier for the encounter place of service	nvarchar	848383005	VISIT_OCCURRENCE	care_site_id		Assign an ID to each combination of a location and nature of the site - the latter could be the Place of Service, name or another characteristic in your source data.	integer	Match	
PATIENT NOTES	service_location_name	Text description of the encounter place of service	nvarchar	Smile Hospital	CARE_SITE	care_site_name	The name of the care_site as it appears in the source data		varchar(255)	Match	
MEDICATION	patient_id	An identifier unique to the patient	nvarchar	121212	DRUG_EXPOSURE	person_id	The PERSON_ID of the PERSON for whom the drug dispensing or administration is recorded. This may be a system generated code.		integer	Match	
MEDICATION	encounter_id	An identifier unique to the encounter	nvarchar	101	DRUG_EXPOSURE	visit_occurrence_id	The Visit during which the drug was prescribed, administered or dispensed.	To populate this field drug exposures must be explicitly initiated in the visit.	integer	Match	
MEDICATION	medication_type	The medication data element source type, e.g., order, administration, prescription, dispensed med, and home med. This is often noted during medication reconciliation.	nvarchar	order	DRUG_EXPOSURE	drug_type_concept_id	You can use the TYPE_CONCEPT_ID to delineate between prescriptions written vs. prescriptions dispensed vs. medication history vs. patient-reported exposure, etc.	Choose the drug_type_concept_id that best represents the provenance of the record, for example whether it came from a record of a prescription written or physician administered drug. Accepted Concepts. A more detailed explanation of each Type Concept can be found on the vocabulary wiki.	integer	Match and Transform	IC: https://athena.ohdsi.org/search-terms/terms?domain=Type+Concept&standardConcept=Standard&page=1&pageSize=15&query=
MEDICATION	medication_code	The standard RxNorm or NDC codes used to identify the medicines	nvarchar	318274	DRUG_EXPOSURE	drug_source_value	This field houses the verbatim value from the source data representing the drug exposure that occurred. For example, this could be an NDC or Gemscript code.	This code is mapped to a Standard Drug Concept in the Standardized Vocabularies and the original code is stored here for reference.	varchar(50)	Match	
MEDICATION	medication_description	Text description of the standard Medication Code	nvarchar	amoxicillin 500 mg	CONCEPT, DRUG_STRENGTH	concept_name, amount_value	concept_name: An unambiguous, meaningful and descriptive name for the Concept., amount_value: The numeric value or the amount of active ingredient contained within the drug product.		varchar(255), float	Match and Transform	IC: We can map the description of the NDC code the CONCEPT table, and also extract the AMOUNT_VALUE (500 mg) to place in the DRUG_STRENGTH TABLE. https://ohdsi.github.io/CommonDataModel/drug_dose.html
MEDICATION	medication_code_set	This column is used to indicate the Medication Code codeset, i.e., RxNorm or NDC.	nvarchar	RxNorm	VOCABULARY	vocabulary_id	A unique identifier for each Vocabulary, such as ICD9CM, SNOMED, Visit.		varchar(20)	Match	IC: Should be vocabulary_id based on this: https://www.ohdsi.org/web/wiki/doku.php?id=documentation:cdm:vocabulary
MEDICATION	instructed_start_date_time	Date and time that the patient was instructed to start taking the medication	datetime	2019-11-15 16:20:00	DRUG EXPOSURE	drug_exposure_start_datetime		This is not required, though it is in v6. If a source does not specify datetime the convention is to set the time to midnight (00:00:0000)	datetime	Match and Transform	
MEDICATION	instructed_stop_date_time	Date and time that the patient was instructed to stop taking the medication	datetime	2019-11-19 16:20:00	DRUG EXPOSURE	drug_exposure_end_datetime		This is not required, though it is in v6. If a source does not specify datetime the convention is to set the time to midnight (00:00:0000)	datetime	Match and Transform	
MEDICATION	ordered_date_time	Date and time when the medication was ordered for the patient	datetime	2019-11-15 16:20:00	DRUG EXPOSURE	drug_exposure_start_datetime		This is not required, though it is in v6. If a source does not specify datetime the convention is to set the time to midnight (00:00:0000)	datetime	Match and Transform	
MEDICATION	administered_date_time	Date and time that the medication administration was documented in the system	datetime	2019-11-15 16:30:00	DRUG EXPOSURE	drug_exposure_start_datetime, drug_exposure_end_datetime, verbatim_end_date	verbatim_end_date: This is the end date of the drug exposure as it appears in the source data, if it is given	drug_exposure_start_datetime/drug_exposure_end_datetime: This is not required, though it is in v6. If a source does not specify datetime the convention is to set the time to midnight (00:00:0000) verbatim_end_date: Put the end date or discontinuation date as it appears from the source data or leave blank if unavailable.	datetime, datetime, date	Match and Transform	IC: If drug is an administered drug, end date = start date. Since we are making a guess/assumption, we have to set verbatim_end_date to NULL https://forums.ohdsi.org/t/drug-exposure-quantity-recalculation/9607/7, https://forums.ohdsi.org/t/drug-exposure-end-date/3401/5. We would need to choose the drug_type_concept_id that matches administered drug

MEDICATION	medication_documentation_date_time	Date and time when the patient was on the medication prior to entering the ED	datetime	2019-11-15 16:30:00	DRUG EXPOSURE	drug_exposure_start_datetime		This is not required, though it is in v6. If a source does not specify datetime the convention is to set the time to midnight (00:00:0000)	datetime	Match and Transform	
MEDICATION	dose_quantity	This is the quantity of dose prescribed to the patient	nvarchar	1 capsule	DRUG EXPOSURE, DRUG STRENGTH, RELATIONSHIP	quantity, sig		To find the dose form of a drug the RELATIONSHIP table can be used where the relationship_id is 'Has dose form'. If liquid, quantity stands for the total amount dispensed or ordered of ingredient in the units given by the drug_strength table. If the unit from the source data does not align with the unit in the DRUG_STRENGTH table the quantity should be converted to the correct unit given in DRUG_STRENGTH. For clinical drugs with fixed dose forms (tablets etc.) the quantity is the number of units/tablets/capsules prescribed or dispensed (can be partial, but then only 1/2 or 1/3, not 0.01). Clinical drugs with divisible dose forms (injections) the quantity is the amount of ingredient the patient got. For example, if the injection is 2mg/mL but the patient got 80mL then quantity is reported as 160. Quantified clinical drugs with divisible dose forms (prefilled syringes), the quantity is the amount of ingredient similar to clinical drugs. Please see how to calculate drug dose for more information.	float	Match and Transform	IC: Looks very tricky, we will need to use multiple tables to autopopulate this.We can also combine this column with dose_frequency to create the sig column. See dose_frequency for more details on sig
MEDICATION	dose_quantity_unit	Description for Dose Quantity Unit	nvarchar	g	DRUG_EXPOSURE	dose_unit_source_value	This field houses the verbatim value from the source data representing the dose unit of the drug given.	This information may be called something different in the source data but the field is meant to contain a value indicating the unit of dosage of drug given to the patient. This is an older column and will be deprecated in an upcoming version.	varchar(50)	Match	IC: Note will be deprecated soon
MEDICATION	dose_frequency	Frequency of the medication dose	nvarchar	One time a day	DRUG EXPOSURE	sig	This is the verbatim instruction for the drug as written by the provider.	Put the written out instructions for the drug as it is verbatim in the source, if available.	nvarchar	Match and Transform	IC: use this column and the dose_quantity to create this free text instructions field
MEDICATION	route_code	Codes that indicate the path by which a medication is administered into the body	nvarchar	C38288	DRUG_EXPOSURE				nvarchar	No equivalent in OMOP	
MEDICATION	route_description	Text description of the Route Code	nvarchar	oral	DRUG_EXPOSURE	route_source_value	This field houses the verbatim value from the source data representing the drug route.	This information may be called something different in the source data but the field is meant to contain a value indicating when and how a drug was given to a patient. This source value is mapped to a standard concept which is stored in the ROUTE_CONCEPT_ID field.	varchar(50)	Match	
MEDICATION	service_provider_npi	The unique NPI for the clinician providing the encounter services	nvarchar	5555555553	PROVIDER	npi	This is the National Provider Number issued to health care providers in the US by the Centers for Medicare and Medicaid Services (CMS).		varchar(20)	Match	
MEDICATION	service_provider_first_name	First name of the clinician providing the billed encounter services	nvarchar	Ahmed	PROVIDER	provider_name		This field is not necessary as it is not necessary to have the actual identity of the Provider. Rather, the idea is to uniquely and anonymously identify providers of care across the database.	varchar(255)	Match and Transform	IC: Need to combine first_name and last_name
MEDICATION	service_provider_last_name	Last name of the clinician providing the billed encounter services	nvarchar	Mahmood	PROVIDER	provider_name		This field is not necessary as it is not necessary to have the actual identity of the Provider. Rather, the idea is to uniquely and anonymously identify providers of care across the database.	varchar(255)	Match and Transform	IC: Need to combine first_name and last_name
MEDICATION	service_location_id	Unique identifier for the encounter place of service	nvarchar	848383005	VISIT_OCCURRENCE	care_site_id		Assign an ID to each combination of a location and nature of the site - the latter could be the Place of Service, name or another characteristic in your source data.	integer	Match	
MEDICATION	service_location_name	Text description of the encounter place of service	nvarchar	Smile Hospital	CARE_SITE	care_site_name	The name of the care_site as it appears in the source data		varchar(255)	Match	
FAMILY HISTORY	patient_id	An identifier unique to the patient	nvarchar	121212	OBSERVATION	person_id	The PERSON_ID of the Person for whom the Observation is recorded. This may be a system generated code.		integer	Match	
FAMILY HISTORY	encounter_id	An identifier unique to the encounter	nvarchar	101	OBSERVATION	visit_occurrence_id	The visit during which the Observation occurred.	Depending on the structure of the source data, this may have to be determined based on dates. If an OBSERVATION_DATE occurs within the start and end date of a Visit it is a valid ETL choice to choose the VISIT_OCCURRENCE_ID from the visit that subsumes it, even if not explicitly stated in the data. While not required, an attempt should be made to locate the VISIT_OCCURRENCE_ID of the observation record. If an observation is related to a visit explicitly in the source data, it is possible that the result date of the Observation falls outside of the bounds of the Visit dates.	integer	Match	
FAMILY HISTORY	family_history_type_code	SNOMED CT code representing family medical history hierarchy	nvarchar	266959005	OBSERVATION	observation_source_value	This field houses the verbatim value from the source data representing the Observation that occurred. For example, this could be an ICD10 or Read code.	This code is mapped to a Standard Concept in the Standardized Vocabularies and the original code is stored here for reference.	varchar(50)	Match	
FAMILY HISTORY	family_history_type_description	Text description for Family History Type Code	nvarchar	Grandparent has no history of colon cancer	CONCEPT	concept_name	An unambiguous, meaningful and descriptive name for the Concept.		varchar(255)	Match	
FAMILY HISTORY	family_history_observed_value	Unstructured/non-standard family history documentation	nvarchar	no cancer in family	OBSERVATION	value_as_string	This is the categorical value of the Result of the Observation, if applicable and available.		varchar(60)	Match	
FAMILY HISTORY	family_history_documentation_date_time	Date and time when the family history is recorded	datetime	2019-11-16 10:20:00	OBSERVATION	observation_datetime		If no time is given set to midnight (00:00:00).	datetime	Match	
FAMILY HISTORY	service_provider_npi	The unique NPI for the clinician note recorder. If the note recorder does not have an NPI, leave this column blank.	nvarchar	5555555553	PROVIDER	npi	This is the National Provider Number issued to health care providers in the US by the Centers for Medicare and Medicaid Services (CMS).		varchar(20)	Match	
FAMILY HISTORY	service_provider_first_name	First name of the note recorder	nvarchar	Ahmed	PROVIDER	provider_name		This field is not necessary as it is not necessary to have the actual identity of the Provider. Rather, the idea is to uniquely and anonymously identify providers of care across the database.	varchar(255)	Match and Transform	IC: Need to combine first_name and last_name
FAMILY HISTORY	service_provider_last_name	Last name of the note recorder	nvarchar	Pesari	PROVIDER	provider_name		This field is not necessary as it is not necessary to have the actual identity of the Provider. Rather, the idea is to uniquely and anonymously identify providers of care across the database.	varchar(255)	Match and Transform	IC: Need to combine first_name and last_name
FAMILY HISTORY	onset_date	Documented date that the family member's problem began	date	2020-11-17	OBSERVATION	obvservation_date, concept_id	The date of the Observation. Depending on what the Observation represents this could be the date of a lab test, the date of a survey, or the date a patient's family history was taken.	For some observations the ETL may need to make a choice as to which date to choose.	date	Match and Transform	IC: Need to create a Concept_id for "date of onset" (4181873 Link between the diagnosis record in CONDITION_OCCURENCE table and the date of onset record in OBSERVATION table should be established via FACT_RELATIONSHIP table See more here: https://forums.ohdsi.org/t/how-to-store-date-of-onset-and-date-of-diagnosis-for-a-disease/9460
FAMILY HISTORY	service_location_id	Unique identifier for the encounter place of service	nvarchar	848383005	VISIT_OCCURRENCE	care_site_id		Assign an ID to each combination of a location and nature of the site - the latter could be the Place of Service, name or another characteristic in your source data.	integer	Match	
FAMILY HISTORY	service_location_name	Text description of the encounter place of service	nvarchar	Smile Hospital	CARE_SITE	care_site_name	The name of the care_site as it appears in the source data		varchar(255)	Match	
SOCIAL HISTORY	patient_id	An identifier unique to the patient	nvarchar	121212	OBSERVATION	person_id	The PERSON_ID of the Person for whom the Observation is recorded. This may be a system generated code.		integer	Match	
SOCIAL HISTORY	encounter_id	An identifier unique to the encounter	nvarchar	101	OBSERVATION	visit_occurrence_id	The visit during which the Observation occurred.	Depending on the structure of the source data, this may have to be determined based on dates. If an OBSERVATION_DATE occurs within the start and end date of a Visit it is a valid ETL choice to choose the VISIT_OCCURRENCE_ID from the visit that subsumes it, even if not explicitly stated in the data. While not required, an attempt should be made to locate the VISIT_OCCURRENCE_ID of the observation record. If an observation is related to a visit explicitly in the source data, it is possible that the result date of the Observation falls outside of the bounds of the Visit dates.	integer	Match	
SOCIAL HISTORY	social_history_type_code	Standard code for social history (e.g. SNOMED where applicable)	nvarchar	266919005	OBSERVATION	observation_source_value	This field houses the verbatim value from the source data representing the Observation that occurred. For example, this could be an ICD10 or Read code.	This code is mapped to a Standard Concept in the Standardized Vocabularies and the original code is stored here for reference.	varchar(50)	Match	
SOCIAL HISTORY	social_history_type_description	Text description for Social History Type Code	nvarchar	Patient Never smoked	CONCEPT	concept_name	An unambiguous, meaningful and descriptive name for the Concept.		varchar(255)	Match	
SOCIAL HISTORY	social_history_observed_value	Unstructured/non-standard social history documentation	nvarchar	non_smoker	OBSERVATION	value_as_string	This is the categorical value of the Result of the Observation, if applicable and available.		varchar(60)	Match	
SOCIAL HISTORY	social_history_observed_value_description	Free text description for Social History Observed Value	nvarchar	patient does not smoke	OBSERVATION	value_source_value	This field houses the verbatim result value of the Observation from the source data. Do not get confused with the Observation_source_value which captures source value of the observation mapped to observation_concept_id. This field is the observation result value from the source.	If the observation_source_value was a question, for example, or an observation that requires a result then this field is the answer/ result from the source data. Store the verbatim value that represents the result of the observation_source_value.	varchar(50)	Match	
SOCIAL HISTORY	social_history_documentation_date_time	Date and time the social history was documented	datetime	2019-12-19 11:00:00	OBSERVATION	observation_datetime		If no time is given set to midnight (00:00:00).	datetime	Match	
SOCIAL HISTORY	effective_start_date	Start date of the social history element noted by the patient	date	2019-12-17	OBSERVATION	observation_date	The date of the Observation. Depending on what the Observation represents this could be the date of a lab test, the date of a survey, or the date a patient's family history was taken.	For some observations the ETL may need to make a choice as to which date to choose.	date	Match and Transform	IC: Its very hard to distiguish between documentation date/onset date/start date in OMOP. https://forums.ohdsi.org/t/time-of-observation-vs-time-observation-happened/12126
SOCIAL HISTORY	effective_stop_date	Date that the social history element was resolved or stopped	date	2019-12-18	OBSERVATION	observation_date	The date of the Observation. Depending on what the Observation represents this could be the date of a lab test, the date of a survey, or the date a patient's family history was taken.	For some observations the ETL may need to make a choice as to which date to choose.	date	Match and Transform	IC: Its very hard to distiguish between documentation date/onset date/start date in OMOP. https://forums.ohdsi.org/t/time-of-observation-vs-time-observation-happened/12126
SOCIAL HISTORY	service_provider_npi	The unique NPI of the clinician note recorder. If the note recorder does not have an NPI, leave this column blank.	nvarchar	5555555553	PROVIDER	npi	This is the National Provider Number issued to health care providers in the US by the Centers for Medicare and Medicaid Services (CMS).		varchar(20)	Match	
SOCIAL HISTORY	service_provider_first_name	First name of the note recorder	nvarchar	Ahmed	PROVIDER	provider_name		This field is not necessary as it is not necessary to have the actual identity of the Provider. Rather, the idea is to uniquely and anonymously identify providers of care across the database.	varchar(255)	Match and Transform	IC: Need to combine first_name and last_name
SOCIAL HISTORY	service_provider_last_name	Last name of the note recorder	nvarchar	Mahmood	PROVIDER	provider_name		This field is not necessary as it is not necessary to have the actual identity of the Provider. Rather, the idea is to uniquely and anonymously identify providers of care across the database.	varchar(255)	Match and Transform	IC: Need to combine first_name and last_name
SOCIAL HISTORY	service_location_id	Unique identifier for the encounter place of service	nvarchar	848383005	VISIT_OCCURRENCE	care_site_id		Assign an ID to each combination of a location and nature of the site - the latter could be the Place of Service, name or another characteristic in your source data.	integer	Match	
SOCIAL HISTORY	service_location_name	Text description of the encounter place of service	nvarchar	Smile Hospital	CARE_SITE	care_site_name	The name of the care_site as it appears in the source data		varchar(255)	Match	
PLAN OF CARE	patient_id	An identifier unique to the patient	nvarchar	121212	NOTE	person_id			integer	Match	
PLAN OF CARE	encounter_id	An identifier unique to the encounter	nvarchar	101	NOTE	visit_occurrence_id	The Visit during which the note was written.		integer	Match	
PLAN OF CARE	plan_of_care_id	An identifier unique to each documented plan of care	nvarchar	314235	NOTE	note_id	A unique identifier for each note.		integer	Match	
PLAN OF CARE	plan_of_care_instructions	These are instructions given to the patient. This is typically a free text note.	nvarchar(MAX)	Take your medication daily	NOTE	note_text	The content of the note.		varchar(MAX)	Match	
PLAN OF CARE	plan_of_care_effective_date	This is the date of plan of care documentation	date	2019-12-16	NOTE	note_date	The date the note was recorded.		date	Match	
PLAN OF CARE	plan_of_care_code	Standard SNOMED-CT code for plan of care education	nvarchar	225323002	OBSERVATION	observation_source_value	This field houses the verbatim value from the source data representing the Observation that occurred. For example, this could be an ICD10 or Read code.	This code is mapped to a Standard Concept in the Standardized Vocabularies and the original code is stored here for reference.	varchar(50)	Match	
PLAN OF CARE	plan_of_care_description	Text description for Plan of Care Code	nvarchar	High Blood Pressure mitigation	CONCEPT	concept_name	An unambiguous, meaningful and descriptive name for the Concept.		varchar(255)	Match	
PLAN OF CARE	service_location_id	Unique identifier for the encounter place of service	nvarchar	848383005	VISIT_OCCURRENCE	care_site_id		Assign an ID to each combination of a location and nature of the site - the latter could be the Place of Service, name or another characteristic in your source data.	integer	Match	
PLAN OF CARE	service_location_name	Text description of the encounter place of service	nvarchar	Smile Hospital	CARE_SITE	care_site_name	The name of the care_site as it appears in the source data		varchar(255)	Match	
PAYER	patient_id	An identifier unique to the patient	nvarchar	121212	PAYER_PLAN_PERIOD	person_id	The Person covered by the Plan.	A single Person can have multiple, overlapping, PAYER_PLAN_PERIOD records	integer	Match	
PAYER	encounter_id	An identifier unique to the encounter	nvarchar	101						No equivalent in OMOP	IC: No visit_occurrence_id in the PAYER_PLAN_PERIOD table
PAYER	insurance_plan_name	Patient Insurance plan name	nvarchar	ABC Partners Health Plan	PAYER_PLAN_PERIOD	payer_source_value	This is the Payer as it appears in the source data.		varchar(50)	Match	
PAYER	insurance_group_id	Group ID information normally refers to the patient's employer or where they get their insurance coverage from	nvarchar	ANB11111111111	PAYER_PLAN_PERIOD	sponsor_source_value	The Plan sponsor as it appears in the source data.		varchar(50)	Match	
PAYER	insurance_start_date	Start date or effective date of insurance	date	2019-12-17	PAYER_PLAN_PERIOD	payer_plan_period_start_date	Start date of Plan coverage.		date	Match	
PAYER	insurance_end_date	End date of insurance	date	2020-12-17	PAYER_PLAN_PERIOD	payer_plan_period_end_date	End date of Plan coverage.		date	Match	
PAYER	policy_id	The Insurance Policy ID	nvarchar	11111111111	PAYER_PLAN_PERIOD	family_source_value	The common identifier for all people (often a family) that covered by the same policy.	Often these are the common digits of the enrollment id of the policy members.	varchar(50)	Match	
PAYER	insurance_active	Is the Insurance Still Active	boolean	Yes						No equivalent in OMOP	
PAYER	service_location_id	Unique identifier for the encounter place of service	nvarchar	848383005	VISIT_OCCURRENCE	care_site_id		Assign an ID to each combination of a location and nature of the site - the latter could be the Place of Service, name or another characteristic in your source data.	integer	Match	
PAYER	service_location_name	Text description of the encounter place of service	nvarchar	Smile Hospital	CARE_SITE	care_site_name	The name of the care_site as it appears in the source data		varchar(255)	Match	
ALLERGY	patient_id	An identifier unique to the patient	nvarchar	121212	OBSERVATION	person_id	The PERSON_ID of the Person for whom the Observation is recorded. This may be a system generated code.		integer	Match	
ALLERGY	encounter_id	An identifier unique to the encounter	nvarchar	101	OBSERVATION	visit_occurrence_id	The visit during which the Observation occurred.	Depending on the structure of the source data, this may have to be determined based on dates. If an OBSERVATION_DATE occurs within the start and end date of a Visit it is a valid ETL choice to choose the VISIT_OCCURRENCE_ID from the visit that subsumes it, even if not explicitly stated in the data. While not required, an attempt should be made to locate the VISIT_OCCURRENCE_ID of the observation record. If an observation is related to a visit explicitly in the source data, it is possible that the result date of the Observation falls outside of the bounds of the Visit dates.	integer	Match	
ALLERGY	allergy_type_code	A standard code such as SNOMED that indicates the type of allergy	nvarchar	416098002	OBSERVATION	observation_source_value	This field houses the verbatim value from the source data representing the Observation that occurred. For example, this could be an ICD10 or Read code.	This code is mapped to a Standard Concept in the Standardized Vocabularies and the original code is stored here for reference.	varchar(50)	Match and Transform	IC: we would probably need to use the CONCEPT_RELATIONSHIP to nest allergy_type_code and allergy_code together
ALLERGY	allergy_type_description	Text description of the type of allergy (e.g. drug or food)	nvarchar	drug allergy (disorder)	CONCEPT	concept_name	An unambiguous, meaningful and descriptive name for the Concept.		varchar(255)	Match	
ALLERGY	allergic_to_code	A standard code representing what the patient is allergic to where applicable (e.g RxNorm if medication allergy)	nvarchar	722	OBSERVATION	observation_source_value	This field houses the verbatim value from the source data representing the Observation that occurred. For example, this could be an ICD10 or Read code.	This code is mapped to a Standard Concept in the Standardized Vocabularies and the original code is stored here for reference.	varchar(50)	Match and Transform	IC: we would probably need to use the CONCEPT_RELATIONSHIP to nest allergy_type_code and allergy_code together
ALLERGY	allergic_to_description	Text description of what the patient is allergic to where applicable	nvarchar	Amoxicillin	CONCEPT	concept_name	An unambiguous, meaningful and descriptive name for the Concept.		varchar(255)	Match	
ALLERGY	allergic_reaction	Text description of patient's allergic reaction	nvarchar	patient breaks out into hives after taking amoxicillin	OBSERVATION	value_as_string	e categorical value of the Result of the Observation, if applicable and available.		varchar(60)	Match	IC: We can combine allergic_reaction_description and allergic_reaction together
ALLERGY	allergic_reaction_description	Additional free text description of patient's allergic reaction [optional]	nvarchar	Severe hives on arms	OBSERVATION	value_as_string	e categorical value of the Result of the Observation, if applicable and available.		varchar(60)	Match	IC: We can combine allergic_reaction_description and allergic_reaction together
ALLERGY	allergy_start_date	Date the patient's allergy started	date	2018-01-01	OBSERVATION	observation_date	The date of the Observation. Depending on what the Observation represents this could be the date of a lab test, the date of a survey, or the date a patient's family history was taken.	For some observations the ETL may need to make a choice as to which date to choose.	date	Match and Transform	IC: Its very hard to distiguish between documentation date/onset date/start date in OMOP. https://forums.ohdsi.org/t/time-of-observation-vs-time-observation-happened/12126
ALLERGY	allergy_end_date	Date the patient's allergy ended if applicable	date		OBSERVATION	observation_date	The date of the Observation. Depending on what the Observation represents this could be the date of a lab test, the date of a survey, or the date a patient's family history was taken.	For some observations the ETL may need to make a choice as to which date to choose.	date	Match and Transform	IC: Its very hard to distiguish between documentation date/onset date/start date in OMOP. https://forums.ohdsi.org/t/time-of-observation-vs-time-observation-happened/12126
ALLERGY	allergy_status_code	Code that represents whether the allergy status is active or complete	nvarchar	1						No equivalent in OMOP	
ALLERGY	allergy_status_description	Text description of Allergy Status Code	nvarchar	active						No equivalent in OMOP	
ALLERGY	allergy_documentation_date_time	The date and time that the allergy was documented	datetime	2019-12-23 16:35:00	OBSERVATION	observation_datetime		If no time is given set to midnight (00:00:00).	datetime	Match	
ALLERGY	service_location_id	Unique identifier for the encounter place of service	nvarchar	848383005	CARE_SITE	care_site_id		Assign an ID to each combination of a location and nature of the site - the latter could be the Place of Service, name or another characteristic in your source data.	integer	Match	
ALLERGY	service_location_name	Text description of the encounter place of service	nvarchar	Smile Hospital	CARE_SITE	care_site_name	The name of the care_site as it appears in the source data		varchar(255)	Match	
LOCATION	service_location_id	Unique identifier for the encounter place of service	nvarchar	848383005	CARE_SITE	care_site_source_value, care_site_id	The identifier of the care_site as it appears in the source data. This could be an identifier separate from the name of the care_site.			Match and Transform	IC: We use this column to create a care_site_id
LOCATION	service_location_name	Text description of the encounter place of service	nvarchar	Smile Hospital	CARE_SITE	care_site_name	The name of the care_site as it appears in the source data		varchar(255)	Match	
LOCATION	service_location_street_address	Street address of the service location where services were provided	nvarchar	100 Country Road	LOCATION	address_1, address_2	This is the first line of the address., This is the second line of the address		varchar(50), varchar(50)	Match and Transform	IC: We can split the address into two columns or place everything into address_1
LOCATION	service_location_city	City of the service location where services were provided	nvarchar	Dallas	LOCATION	city			varchar(50)	Match	
LOCATION	service_location_state	State of the service location where services were provided	nvarchar	Texas	LOCATION	state			varchar(2)	Match and Transform	IC: We would need to abbreviate the states.
LOCATION	service_location_zip_code	Zip code of the service location where services were provided	nvarchar	75261	LOCATION	zip		Zip codes are handled as strings of up to 9 characters length. For US addresses, these represent either a 3-digit abbreviated Zip code as provided by many sources for patient protection reasons, the full 5-digit Zip or the 9-digit (ZIP + 4) codes. Unless for specific reasons analytical methods should expect and utilize only the first 3 digits. For international addresses, different rules apply.	varchar(9)	Match	
BILLING DATA	patient_id	An identifier unique to the patient, may be the MRN	nvarchar	121212	PERSON	person_id	It is assumed that every person with a different unique identifier is in fact a different person and should be treated independently.	Any person linkage that needs to occur to uniquely identify Persons ought to be done prior to writing this table. This identifier can be the original id from the source data provided if it is an integer, otherwise it can be an autogenerated number.	integer	Match and Transform	IC: Could be either person_id or person_source value depending on whether MRN is used. If MRN used, will need to autogenerate a number
BILLING DATA	patient_mrn	Medical Record Number for the patient, preferably the same identifier used by the emergency department where service was provided	nvarchar	1482928	PERSON	person_source_value	Use this field to link back to persons in the source data. This is typically used for error checking of ETL logic.	Some use cases require the ability to link back to persons in the source data. This field allows for the storing of the person value as it appears in the source. This field is not required but strongly recommended.	varchar(50)	Match	
BILLING DATA	patient_first_name	Patient's First name	nvarchar	Jane						No equivalent in OMOP	IC: We could place it in the source_value column but it is discouraged, or add a column. https://forums.ohdsi.org/t/patient-name-in-omop-db/18449/4
BILLING DATA	patient_last_name	Patient's Last name	nvarchar	Doe						No equivalent in OMOP	IC: We could place it in the source_value column but it is discouraged, or add a column. https://forums.ohdsi.org/t/patient-name-in-omop-db/18449/4
BILLING DATA	patient_gender	Patient's Gender	nvarchar	Female	PERSON	gender_source_value	This field is used to store the biological sex of the person from the source data. It is not intended for use in standard analytics but for reference only.	Put the biological sex of the person as it appears in the source data.	varchar(50)	Match	IC: Need to ensure that it is sex rather than gender
BILLING DATA	patient_date_of_birth	Patient's Date of birth	date	2016-10-15	PERSON	birth_datetime		This field is not required but highly encouraged. For data sources that provide the precise datetime of birth, that value should be stored in this field. If birth_datetime is not provided in the source, use the following logic to infer the date: If day_of_birth is null and month_of_birth is not null then use the first of the month in that year. If month_of_birth is null or if day_of_birth AND month_of_birth are both null and the person has records during their year of birth then use the date of the earliest record, otherwise use the 15th of June of that year. If time of birth is not given use midnight (00:00:0000).	datetime	Match and Transform	IC: Change from date to datetime assume midnight. Will also need to use this field to engineer birth_day, birth_month, and birth_year
BILLING DATA	patient_date_of_death	If patient is deceased, the death date	date		DEATH	death_date	The date the person was deceased.	If the precise date include day or month is not known or not allowed, December is used as the default month, and the last day of the month the default day.	date	Match	
BILLING DATA	policy_group_ID	An identifier unique to the insured's policy group	nvarchar	111112	PAYER_PLAN_PERIOD	sponsor_source_value	The Plan sponsor as it appears in the source data.		varchar(50)	Match	
BILLING DATA	policy_group_name	Text descriptor of the identified group policy	nvarchar	High-deductible, HSA	CONCEPT	concept_name	An unambiguous, meaningful and descriptive name for the Concept.		varchar(255)	Match	
BILLING DATA	policy_plan_ID	An identifier unique to the insured's insurance plan	nvarchar	11111111112	PAYER_PLAN_PERIOD	family_source_value	The common identifier for all people (often a family) that covered by the same policy.	Often these are the common digits of the enrollment id of the policy members.	varchar(50)	Match	
BILLING DATA	policy_plan_name	Text descriptor of the identified insurance plan	nvarchar	United Healthcare	PAYER_PLAN_PERIOD	payer_source_value	This is the Payer as it appears in the source data.		varchar(50)	Match	
BILLING DATA	policy_active_flag	Indicates if the insured's policy is active or inactive	nvarchar	yes						No equivalent in OMOP	
BILLING DATA	service_location_id	Unique identifier for the encounter place of service	nvarchar	848383005	CARE_SITE	care_site_id		Assign an ID to each combination of a location and nature of the site - the latter could be the Place of Service, name or another characteristic in your source data.	integer	Match	
BILLING DATA	service_location_name	Text description of the encounter place of service	nvarchar	Smile Hospital	CARE_SITE	care_site_name	The name of the care_site as it appears in the source data		varchar(255)	Match	
BILLING DATA	encounter_id	Unique identification number for an encounter, preferably the same identifier used by the emergency department where service was provided	nvarchar	1010101	VISIT_OCCURRENCE	visit_occurrence_id	Use this to identify unique interactions between a person and the health care system. This identifier links across the other CDM event tables to associate events with a visit.	This should be populated by creating a unique identifier for each unique interaction between a person and the healthcare system where the person receives a medical good or service over a span of time.	integer	Match	IC: Will need to specify that it is a Case-Management Visit
BILLING DATA	encounter_date_time	Date (and time if available) the encounter/service started	datetime	2021-01-01 18:40:00	VISIT_OCCURRENCE	visit_start_datetime		If no time is given for the start date of a visit, set it to midnight (00:00:0000).	datetime	Match	IC: Will need to specify that it is a Case-Management Visit
BILLING DATA	encounter_discharge_date	Date (and time if available) the encounter ended	datetime	2021-01-01 22:40:00	VISIT_OCCURRENCE	visit_end_datetime	If a Person is still an inpatient in the hospital at the time of the data extract and does not have a visit_end_datetime, then set the visit_end_datetime to the datetime of the data pull.	If no time is given for the end date of a visit, set it to midnight (00:00:0000).	datetime	Match	IC: Will need to specify that it is a Case-Management Visit
BILLING DATA	discharge_disposition_code	Standard code representing the disposition status of the patient	nvarchar	ADM	VISIT_OCCURRENCE	discharged_to_concept_id	Use this field to determine where the patient was discharged to after a visit. This concept is part of the visit domain and can indicate if a patient was transferred to another hospital or sent to a long-term care facility, for example. It is assumed that a person is discharged to home therefore there is not a standard concept id for "home". Use concept id = 0 when a person is discharged to home.	If available, map the discharged_to_source_value to a standard concept in the visit domain.	integer	Match and Transform	IC: Need to check whether the CEDR matches to an accepted concept. https://athena.ohdsi.org/search-terms/terms?domain=Visit&standardConcept=Standard&page=1&pageSize=15&query=
BILLING DATA	discharge_disposition_description	Text description of the coded discharge disposition	nvarchar	Admitted	VISIT_OCCURRENCE	discharged_to_source_value		This information may be called something different in the source data but the field is meant to contain a value indicating where a person was discharged to after a visit, as in they went home or were moved to long-term care. Typically this applies only to visits that have a length of stay of a day or more.	varchar(50)	Match	
BILLING DATA	referring_provider_name	First and last name of provider referring patient for the encounter service [optional]	nvarchar	Linda Smith	PROVIDER	provider_name		This field is not necessary as it is not necessary to have the actual identity of the Provider. Rather, the idea is to uniquely and anonymously identify providers of care across the database.	varchar(255)	Match and Transform	IC: Need to combine first_name and last_name
BILLING DATA	referring_provider_npi	Identifier of the provider referring patient for the encounter service [optional]	nvarchar	5555555555	PROVIDER	npi	This is the National Provider Number issued to health care providers in the US by the Centers for Medicare and Medicaid Services (CMS).		varchar(20)	Match	
BILLING DATA	encounter_diagnosis_code	ICD code(s) representing one or more diagnoses associated to the encounter. Add additional, individual delimited columns as needed to represent all documented ICD codes related to the encounter_id	nvarchar	S09.90	CONDITION_OCCURRENCE	condition_source_value	This field houses the verbatim value from the source data representing the condition that occurred. For example, this could be an ICD10 or Read code.	This code is mapped to a Standard Condition Concept in the Standardized Vocabularies and the original code is stored here for reference.	varchar(50)	Match and Transform	IC: Need to use the condition_type_concept_id to specify that it is a Billing diagnosis
BILLING DATA	resubmission_code	An identifier indicating that the billing claim information is potentially modified	nvarchar	06						No equivalent in OMOP	
BILLING DATA	procedure_code	This column contains a CPT, HCPCS or CPT II code indicating the encounter type and/or performance measurement code. Add additional, individual delimited columns as needed to represent all CPT or HCPCS codes related to the encounter_id. The primary encounter type should be the first of the procedure_code columns.	nvarchar	99283	PROCEDURE_OCCURRENCE	procedure_source_value	This field houses the verbatim value from the source data representing the procedure that occurred. For example, this could be an CPT4 or OPCS4 code.	Use this value to look up the source concept id and then map the source concept id to a standard concept id.	varchar(50)	Match	
BILLING DATA	procedure_code_modifiers	This column contains modifiers to the codes captured in the procedure_code columns. Add additional, individual delimited columns as needed to communicate all relevant modifiers related to the encounter_id.	nvarchar	25	PROCEDURE_OCCURRENCE	modifier_source_value		The original modifier code from the source is stored here for reference.	varchar(50)	Match	
BILLING DATA	encounter_service_charges	The charged amount associated with each CPT or HCPCS code captured in the procedure_code column(s) representing one or more charges associated to the encounter. Add additional, individual delimited columns as needed to represent all documented charges codes related to the encounter_id	float	234.56	COST	total_cost			float	Match	
BILLING DATA	encounter_days_units	The number of days the service covers or units associated to the CPT code. This is found on 24G of CMS form 1500. [optional]	nvarchar	2						No equivalent in OMOP	
BILLING DATA	rendering_provider_npi	The unique NPI number for the billing service provider / clinician	nvarchar	1515151515	PROVIDER	npi	This is the National Provider Number issued to health care providers in the US by the Centers for Medicare and Medicaid Services (CMS).		varchar(20)	Match	
BILLING DATA	rendering_provider_first_name	First name of the billing service provider / clinician	nvarchar	Sam	PROVIDER	provider_name		This field is not necessary as it is not necessary to have the actual identity of the Provider. Rather, the idea is to uniquely and anonymously identify providers of care across the database.	varchar(255)	Match and Transform	IC: Need to combine first_name and last_name
BILLING DATA	rendering_provider_last_name	Last name of the billing service provider / clinician	nvarchar	Hall	PROVIDER	provider_name		This field is not necessary as it is not necessary to have the actual identity of the Provider. Rather, the idea is to uniquely and anonymously identify providers of care across the database.	varchar(255)	Match and Transform	IC: Need to combine first_name and last_name
BILLING DATA	encounter_tin	The federal tax ID number of the billing entity	nvarchar	234512789						No equivalent in OMOP	
BILLING DATA	encounter_total_charge	The sum of charges for the encounter	float	1426.37	COST	total_cost			float	Match	
BILLING DATA	encounter_amount_paid	The actual amount paid for the encounter services	float	1234.67	COST	total_paid			float	Match and Transform	IC: We would need to figure out/create columns for paid_by_payer, paid_by_patient, paid_patient_copay, etc.
BILLING DATA	patient_relationship_to_insured	States the relationship the patient has to the insured person (self, spouse, child, other) [optional]	nvarchar	Self	PERSON, OBSERVATION, FACT_RELATIONSHIP, PERSON					Match and Transform	IC: We would need to relate one person to another using a combination of tables. https://forums.ohdsi.org/t/how-to-represent-family-history/3386/61
BILLING DATA	insured_person_id_number	The identification number of the insured person [optional]	nvarchar	123456789						No equivalent in OMOP	
BILLING DATA	insured_person_first_name	Insured person's first name [optional]	nvarchar	Jane						No equivalent in OMOP	IC: We could place it in the source_value column but it is discouraged, or add a column. https://forums.ohdsi.org/t/patient-name-in-omop-db/18449/4
BILLING DATA	insured_person_last_name	Insured person's last name [optional]	nvarchar	Doe						No equivalent in OMOP	IC: We could place it in the source_value column but it is discouraged, or add a column. https://forums.ohdsi.org/t/patient-name-in-omop-db/18449/4
BILLING DATA	insured_person_gender	Insured person's gender [optional]	nvarchar	Female	PERSON	gender_source_value	This field is used to store the biological sex of the person from the source data. It is not intended for use in standard analytics but for reference only.	Put the biological sex of the person as it appears in the source data.	varchar(50)	Match	IC: Need to ensure that it is sex rather than gender
BILLING DATA	insured_person_date_of_birth	Insured person's date of birth [optional]	date	2016-10-15	PERSON	birth_datetime		This field is not required but highly encouraged. For data sources that provide the precise datetime of birth, that value should be stored in this field. If birth_datetime is not provided in the source, use the following logic to infer the date: If day_of_birth is null and month_of_birth is not null then use the first of the month in that year. If month_of_birth is null or if day_of_birth AND month_of_birth are both null and the person has records during their year of birth then use the date of the earliest record, otherwise use the 15th of June of that year. If time of birth is not given use midnight (00:00:0000).	datetime	Match and Transform	IC: Change from date to datetime assume midnight. Will also need to use this field to engineer birth_day, birth_month, and birth_year
BILLING DATA	patient_condition_related_to_employment	Answers the question is patient's condition related to employment with YES or NO [optional]	boolean	Yes						No equivalent in OMOP	
BILLING DATA	patient_condition_related_to_auto_accident	Answers the question is patient's condition related to an auto accident with YES or NO [optional]	boolean	No						No equivalent in OMOP	
BILLING DATA	patient_condition_related_to_other_accident	Answers the question is patient's condition related to a different accident with YES or NO [optional]	boolean	No						No equivalent in OMOP	
BILLING DATA	claim_codes	NUCC approved condition codes used to identify additional information about the patient's condition or the claim [optional]	nvarchar	W2						No equivalent in OMOP	
BILLING DATA	date_of_current_illness_injury_or_pregnancy	The date of the patient's current illness, injury, or pregnancy [optional]	date	2021-01-01	CONDITION_OCCURRENCE	condition_start_date	Use this date to determine the start date of the condition	Most often data sources do not have the idea of a start date for a condition. Rather, if a source only has one date associated with a condition record it is acceptable to use that date for both the CONDITION_START_DATE and the CONDITION_END_DATE.	date	Match	
BILLING LOCATION	service_location_id	Unique identifier for the encounter place of service	nvarchar	848383005	CARE_SITE	care_site_source_value, care_site_id	The identifier of the care_site as it appears in the source data. This could be an identifier separate from the name of the care_site.			Match and Transform	IC: We use this column to create a care_site_id
BILLING LOCATION	service_location_name	Text description of the encounter place of service	nvarchar	Smile Hospital	CARE_SITE	care_site_name	The name of the care_site as it appears in the source data		varchar(255)	Match	
BILLING LOCATION	service_location_street_address	Street address of the service location where services were provided	nvarchar	100 Country Road	LOCATION	address_1, address_2	This is the first line of the address., This is the second line of the address		varchar(50), varchar(50)	Match and Transform	IC: We can split the address into two columns or place everything into address_1
BILLING LOCATION	service_location_city	City of the service location where services were provided	nvarchar	Dallas	LOCATION	city			varchar(50)	Match	
BILLING LOCATION	service_location_state	State of the service location where services were provided	nvarchar	Texas	LOCATION	state			varchar(2)	Match and Transform	IC: We would need to abbreviate the states.
BILLING LOCATION	service_location_zip_code	Zip code of the service location where services were provided	nvarchar	75261	LOCATION	zip		Zip codes are handled as strings of up to 9 characters length. For US addresses, these represent either a 3-digit abbreviated Zip code as provided by many sources for patient protection reasons, the full 5-digit Zip or the 9-digit (ZIP + 4) codes. Unless for specific reasons analytical methods should expect and utilize only the first 3 digits. For international addresses, different rules apply.	varchar(9)	Match	
